# Metastatic colorectal cancer cells maintain the TGFβ program and use TGFBI to fuel angiogenesis

**DOI:** 10.7150/thno.51507

**Published:** 2021-01-01

**Authors:** Barbara Chiavarina, Brunella Costanza, Roberto Ronca, Arnaud Blomme, Sara Rezzola, Paola Chiodelli, Ambre Giguelay, Guillame Belthier, Gilles Doumont, Gaetan Van Simaeys, Simon Lacroix, Takehiko Yokobori, Bilguun Erkhem-Ochir, Patrick Balaguer, Vincent Cavailles, Eric Fabbrizio, Emmanuel Di Valentin, Stephanie Gofflot, Olivier Detry, Guy Jerusalem, Serge Goldman, Philippe Delvenne, Akeila Bellahcène, Julie Pannequin, Vincent Castronovo, Andrei Turtoi

**Affiliations:** 1 Cancer Research Institute of Montpellier, Tumor Microenvironment and Resistance to Treatment Laboratory, INSERM U1194, Montpellier, France.; 2 Institut National de la Santé et de la Recherche Médicale, Montpellier, France.; 3 Institut du Cancer de Montpellier, Montpellier, France.; 4 Université de Montpellier, Montpellier, France.; 5 Metastasis Research Laboratory, GIGA Cancer, University of Liège, Liège, Belgium.; 6 University of Brescia, Department of Molecular and Translational Medicine, Brescia, Italy.; 7 Cancer Research Institute of Montpellier, Cancer Bioinformatics and Systems Biology Team, INSERM U1194, Montpellier, France.; 8 Institut de Génomique Fonctionnelle, Montpellier, France.; 9 Centre National de la Recherche Scientifique, Montpellier, France.; 10 Center for Microscopy and Molecular Imaging (CMMI), Université libre de Bruxelles (ULB), rue Adrienne Bolland 8, B-6041 Charleroi (Gosselies), Belgium.; 11 Nuclear Medicine department, ULB Hôpital Érasme, route de Lennik 808, B-1070 Brussels, Belgium.; 12 Gunma University Initiative for Advanced Research, International Open Laboratory, Universities of Liege and Montpellier Laboratory, Gunma University, Gunma, Japan.; 13 Cancer Research Institute of Montpellier, Hormone Signaling and Cancer Laboratory, Montpellier, France.; 14 Cancer Research Institute of Montpellier, Oncogenic Pathways in Cancer Laboratory, INSERM U1194, Montpellier, France.; 15 GIGA-Viral Vectors Platform, University of Liège, Liège, Belgium.; 16 BIOTHEQUE, University of Liege, Liege, Belgium.; 17 Department of Abdominal Surgery, University Hospital, University of Liège, Liège, Belgium.; 18 Department of Medical Oncology, University Hospital, University of Liège, Liège, Belgium.; 19 Department of Pathology, University Hospital, University of Liège, Liège, Belgium.

**Keywords:** alternative TGFβ signaling, liver metastases, endothelial cells

## Abstract

Colorectal cancer (CRC) cells are traditionally considered unresponsive to TGFβ due to mutations in the receptors and/or downstream signaling molecules. TGFβ influences CRC cells only indirectly via stromal cells, such as cancer-associated fibroblasts. However, CRC cell ability to directly respond to TGFβ currently remains unexplored. This represents a missed opportunity for diagnostic and therapeutic interventions.

**Methods:** We examined whether cancer cells from primary CRC and liver metastases respond to TGFβ by inducing TGFβ-induced protein ig-h3 (TGFBI) expression, and the contribution of canonical and non-canonical TGFβ signaling pathways to this effect. We then investigated *in vitro* and *in vivo* TGFBI impact on metastasis formation and angiogenesis. Using patient serum samples and an orthotopic mouse model of CRC liver metastases we assessed the diagnostic/tumor targeting value of novel antibodies against TGFBI.

**Results:** Metastatic CRC cells, such as circulating tumor cells, directly respond to TGFβ. These cells were characterized by the absence of TGFβ receptor mutations and the frequent presence of p53 mutations. The pro-tumorigenic program orchestrated by TGFβ in CRC cells was mediated through TGFBI, the expression of which was positively regulated by non-canonical TGFβ signaling cascades. TGFBI inhibition was sufficient to significantly reduce liver metastasis formation *in vivo*. Moreover, TGFBI pro-tumorigenic function was linked to its ability to stimulate angiogenesis. TGFBI levels were higher in serum samples from untreated patients with CRC than in patients who were receiving chemotherapy. A radiolabeled anti-TGFBI antibody selectively targeted metastatic lesions *in vivo*, underscoring its diagnostic and therapeutic potential.

**Conclusions:** TGFβ signaling in CRC cells directly contributes to their metastatic potential and stromal cell-independence. Proteins downstream of activated TGFβ, such as TGFBI, represent novel diagnostic and therapeutic targets for more specific anti-metastatic therapies.

## Introduction

Colorectal cancer (CRC) is the third most frequent cause of cancer-related death worldwide, usually due to the presence of liver metastases. Unfortunately, 40% of patients with CRC have metastatic lesions in liver already at diagnosis 1. Therefore, metastatic disease management is the most important aspect of CRC care. Despite decades of research, the process of tumor dissemination is insufficiently understood, thus precluding the development of metastasis-specific treatments. It is clear that only a subpopulation of tumor cells is responsible for metastasis formation, and circulating tumor cells (CTC) are considered the most probable actors 2, 3. To make the issue more complex, the tumor microenvironment (TME) also plays an important role. TME cells have been traditionally considered as cancer cell accomplices 4; however, it is now clear that TME is also an important barrier to tumor development 5. Early metastatic cells arrive in a hostile environment and therefore, they might display high plasticity, especially until the establishment of a tumor-promoting paracrine crosstalk with the stroma components.

TGFβ and its signaling machinery are important and if not the most essential mediators of cancer cell plasticity 6. Paradoxically, TGFβ signaling switches from tumor suppressing to tumor promoting activity, particularly during CRC progression. Initially, TGFβ signaling in colon epithelial cells synergizes with the NOTCH pathway to counteract APC mutation-induced Wnt hyperactivation 7. It is widely accepted that for transformation, APC-mutated cells must become unresponsive to TGFβ signaling, and this is often achieved through SMAD4 or/and TGFβ receptor mutations. However, TGFβ also stimulates CRC progression mainly through cancer-associated fibroblasts (CAF) 8 that supply a plethora of TGFβ-induced factors, such as MMPs, PDGF, CTGF, required for CRC cell survival, proliferation and invasion 9, 10. Most CRC cells certainly require the paracrine interaction with CAF. Consequently, the inability to respond to TGFβ stimuli would make CRC metastatic cells particularly vulnerable because they would not benefit from the TGFβ-orchestrated autocrine signaling to adapt to the new environment. Therefore, it could be asked whether some CRC cells harboring inactivating mutations in components of the canonical TGFβ signaling cascade maintain TGFβ responsiveness, for instance via alternative SMAD-independent pathways (e.g. ERK, p38) that are intact in most cancer cell types 9, 10. Moreover, TGFβ receptor mutations do not fully hinder downstream signaling. Indeed, it has been demonstrated that despite the presence of some frameshift mutations, TGFβ receptor type II (TGFBR2) is functional 11. Nonetheless, TGFβ-responsive cancer cells are certainly a minority of all CRC cells and their existence and phenotype remain debatable and unknown.

To gain insights into TGFβ-mediated pro-metastatic activity in CRC cells, we investigated the expression pattern and function of TGFβ-induced protein ig-h3 (TGFBI), a major TGFβ-induced protein. *TGFBI* was identified as a TGFβ-inducible gene in lung adenocarcinoma cells 12. TGFBI contains four FAS1 domains that are thought to be cell adhesion domains conserved between plants and animals 13. The fourth FAS1 domain of TGFBI also contains an RGD motif with strong affinity for integrins 14. Many data exist on TGFBI function in solid cancers, most of which have been recently reviewed by Yokobori & Nishiyama 15. Yet, there is no consensus on whether and in which contexts TGFBI acts as a pro- or anti-tumorigenic molecule. For example, Zhang et al. 16 showed that *Tgfbi*-/- mice rapidly develop lung and liver malignancies. Conversely, Ma et al. 17 found that TGFBI overexpression in CRC cells promotes liver metastasis formation. Paradoxically, very few studies on TGFBI have connected its function to TGFβ activity. However, as TGFBI is modulated by TGFβ signaling, its tumor promoting or suppressing function could be cell- and context-dependent. Here, we found that TGFBI produced by CRC cells following their stimulation by TGFβ promotes angiogenesis, and therefore has a pro-metastatic function. This finding identifies TGFBI as a novel therapeutic target downstream of TGFβ, thus eliminating the need to target the entire pathway and allowing focusing on specific, metastasis-promoting components.

## Materials and methods

### Human samples

The Liege University Hospital ethics committee approved the use of human material for this study. All samples were obtained from the institutional Biobank. Patients were informed that the remaining material could be used for research purposes and consent was presumed as long as they did not oppose (opting-out in line with Belgian law). For TGFBI serum level measurement, 15 healthy individuals and 17 patients with CRC were enrolled. Treatment information and patient status are given in **Table S1**. For immunohistochemistry (IHC) analysis, 78 primary CRC (CRC) samples (**Table S2**) and 21 CRC liver metastasis (CRC-LM) samples (**Table S3**) were included. When available, adjacent normal tissue samples were evaluated as controls.

### Immunohistochemistry

Tissue sections (5-µm thick) were prepared from formalin-fixed paraffin-embedded (FFPE) CRC and CRC-LM samples. Sections were deparaffinized three times in xylene for 5 min, and hydrated through a methanol gradient (100%, 95%, 70%, and 50%). Unspecific peroxidase activity was blocked by incubation in 3% H_2_O_2_/90% methanol for 30 min. After the antigen retrieval step (citrate buffer pH6, 95 °C, 40 min), sections were incubated with Protein Block Serum-Free solution (Protein Block Serum-Free Ready-to-Use, catalog no. X0909, Dako, Glostrup, Denmark) at room temperature (RT) for 30 min, and then with an anti-TGFBI antibody (1:200 dilution; Cell Signaling, Danvers, USA; catalog no. 2719) at 4 °C overnight. Next, samples were washed in PBS, and incubated with Histofine MaxPo-Multi HRP-polymer (Nichirei, Tokyo, Japan; cat. no. 414152F) for 30 min. Sections were washed in PBS three times for 5 min and then stained with 3,3'-diaminobenzidine solution (Agilent-Dako, cat. no. GV800). After counterstaining with hematoxylin (Sigma Aldrich, cat. no. MHS32), they were mounted with Eukitt (Orsatech GmbH, Bobingen, Germany). TGFBI expression was scored in accordance with the previously published methodology 18. Briefly, for each section, staining intensity was evaluated using the following scale: 0 = no staining, 1 = weak, 2 = moderate, and 3 = strong. Then, staining extent (i.e. percentage of the positive area relative to the total section) was quantified as follows: 0 = 0%-25%, 1 = 25%-50%, 2 = 50%-75%, and 3 = 75%-100%. The staining intensity and extent scores were multiplied to yield a composite value, called 'IHC score'. Photographs of representative fields were taken under a Leica DM1000 light microscope (Leica, Wetzlar, Germany) and a Leica MC170 HD camera system. Two independent pathologists scored the samples and the mean scores were reported.

### Immunofluorescence

The paraffin removal, antigen retrieval and blocking steps were performed as described above. Then, sections were washed three times in Tris-buffered saline with 0.05% Tween-20 (TBS-T) and incubated with an anti-TGFBI antibody (1:200 dilution; Cell Signaling, Danvers, USA; catalog no. 2719) at 4 °C overnight. Following five washes in TBS-T (5 min/each), slides were incubated with Histofine MaxPo-Multi HRP-polymer at RT for 30 min. After five washes in TBS-T, the signal was revealed using 100 μL of stain solution (2 μL Opal dye and 98 μL Amplifying Buffer) (Perkin Elmer, Waltham, MA, USA; cat. no. NEL810001KT). Following a 10 min incubation and washes in TBS-T, microwave-assisted antibody removal was performed as described by the manufacturer. After a new blocking step, sections were incubated with anti-vimentin (Cell Signaling; catalog no. 57415) and anti-pan-cytokeratin (Abcam; catalog no. ab24647) antibodies (1:1000 dilution) at 4 °C overnight. After staining with the Opal dye, slides were washed and mounted with VECTASHIELD® Antifade Mounting Medium with DAPI (Vector, Burlingame, USA). Images were captured with a Zeiss Apotome microscope (Zeiss, Oberkochen, Germany).

### Cell culture

The LS174T, LOVO, HT29 and HCT116 CRC cell lines were obtained from ATCC (Virginia, USA). SW1222 CRC cells were a kind gift by Prof. W. Bodmer, Department of Medical Oncology, Weatherall Institute, Oxford, UK. HT29 cells with low and high metastatic potential (HT29lm and HT29hm, respectively) were a kind gift of Dr. Raffaella Giavazzi, Institute Mario Negri, Milano, Italy. CCD-18Co human normal colon fibroblasts were from ATCC. All cell lines were cultured in Dulbecco's Modified Eagle medium (DMEM) supplemented with 10% fetal bovine serum (FBS) (Gibco, Invitrogen, Life Technologies, Carlsbad, CA, USA) at 37 °C in 5% CO_2_ atmosphere. The *TGFBI*-silenced SW1222 cell line was generated using anti-*TGFBI* shRNA-expressing lentiviral particles. Anti-*TGFBI* shRNAs were from Sigma Aldrich (St. Louis, MO, USA; cat. no. TRCN0000062177 (#1) and TRCN0000062175 (#2)). Control shRNA (shNT) was an anti-eGFP shRNA plasmid (Sigma; cat. no. SHC005). All shRNAs were inserted in the pLenti6/V5 vector using the pLenti6/V5 Directional TOPO® Cloning Kit (Invitrogen, Carlsbad, CA, USA, Part # K4955-00). ShRNA-expressing lentiviral vectors were co-transfected in Lenti-X™ 293T cells (Clontech, Mountain View, CA, USA; Part # 632180) with the pLenti6-Luciferase, psPAX2 (Addgene, Cambridge, MA, USA; Part #12260) and pVSV-G plasmids. Viral supernatants were collected at 48 h - 96 h post-transfection, and filtered (0.45 µm). SW1222 cells were incubated with these lentiviral particles for 48h and then selected by incubation with 1 µg/mL puromycin (Sigma Aldrich, St. Louis, MO, USA). Primary human umbilical vein endothelial cells (HUVECs) were used at early passages (passages II-V), and grown on plastic surface coated with porcine gelatin in M199 medium (Invitrogen, Carlsbad, CA, USA) supplemented with 20% fetal calf serum (FCS) (Invitrogen), 100 µg/mL endothelial cell growth factors (Sigma-Aldrich, St. Louis, MO, USA), and 100 µg/mL porcine heparin (Sigma-Aldrich, St. Louis, MO, USA). CTCs and primary cancer cells (CPP) from primary and metastatic CRC biopsies were isolated, and established as previously described 19, 20. They were maintained in ultralow attachment 24-well plates (Corning) with 1 mL of M12 medium that included DMEM-F12 (Gibco), 2 mmol/L of L-glutamine (Gibco, Thermo Fisher Sci., Waltham, MA, USA), 100 unit/mL of penicillin and streptomycin (Gibco, Thermo Fisher Sci., Waltham, MA, USA), N2 supplement (Gibco, Thermo Fisher Sci., Waltham, MA, USA), 20 ng/mL of epidermal growth factor (R&D Systems, Minneapolis, MN, USA) and 10 ng/mL of fibroblast growth factor-basic (R&D Systems, Minneapolis, MN, USA).

Conditioned medium (CM) from CRC cell lines was obtained after 48h incubation of 80% confluent cells in serum-free medium. CM were collected, centrifuged at 150×g, RT, for 5 min, and then added to CCD-18Co cell monolayers (cells were pre-starved in serum-free medium for 6h) for 48h. Then, fibroblast monolayers were washed with PBS twice and lysed for western blot analysis. For incubation with recombinant human TGF-β1 (Roche, catalog no. 11412272001), 80% confluent cells were starved in serum-free medium for 16h and then incubated with 5 ng/ml of recombinant TGF-β1 in serum-free medium for 48 h. Medium with TGF-β1 was refreshed after 24 h.

Human anti-*SMAD2* siRNA (ON-TARGETplus SMARTpool Human SMAD2 (4087)) and scramble siRNA (ON-TARGETplus NonTargeting Control Pool, catalog no. D-001810-10-05) were from Dharmacon. SW1222 cells were transfected with 20 nM of each siRNA using Lipofectamine (Lipofectamine 2000 reagent, catalog no. 11668-019, Life Technologies, Carlsbad, CA, USA).

When indicated, the following compounds were used: SB202190 (5 µM, catalog no. S7067, Sigma-Aldrich), BAY11-7082 (5 µM, catalog no. B5556, Sigma-Aldrich), SP600125 (5 µM, catalog no. S5567, Sigma-Aldrich), MK2206 (1 µM, catalog no. 1032350-13-2, Santa Cruz Biotechnology, Dallas, TX, USA), PD98059 (5 µM, catalog no. 19-143, Merck Millipore, Burlington, MA, USA), ARRY-614 (10 µM, catalog no. S7799, Selleckchem), and LY2228820 (5 µM, catalog no. A413122, Sigma-Aldrich).

### Cell line mutation analysis

The mutational status of the different commercial CRC cell lines was derived from the publicly available COSMIC database (https://cancer.sanger.ac.uk/cosmic). The mutation status of CTC44 and CTC45 cells was extracted from the previously published and deposited RNAseq data (BioProject no. PRJNA384289).

### Western blot analysis

Crushed snap-frozen tissue samples and cell pellets were lysed in RIPA buffer (150 mM NaCl, 0.5% Na-deoxycholate, 1% Triton X-100, 0.5% SDS, 50 mM Tris-HCl (pH 7.5)) and protease/phosphatase inhibitor cocktails (catalog no. 16829900; Sigma-Aldrich). Protein lysates were quantified using the Pierce BCA Protein Assay Kit (Thermo Scientific; catalog no. 23225). Laemmli buffer (0.1% 2-mercaptoethanol, 0.0005% bromophenol blue, 10% glycerol, 2% SDS in 63 mM Tris-HCl (pH 6.8)) was added to 20 μg of protein extracts that were then boiled for 5 min and loaded on 10% polyacrylamide gels. Proteins were transferred to nitrocellulose membranes at 100 V for 2h. After blocking in 5% skim milk, membranes were incubated (4 °C, overnight) with antibodies against TGFBI (1:500; Cell Signaling; catalog no. 2719), SMAD2/3 (1:1000; Cell Signaling; catalog no. 8685,) and beta actin, used as loading control (1:10000; Cell Signaling; catalog no. 4967).

### *In vitro* HUVEC-based assays

CRC cells (50,000/cm^2^) were seeded in complete medium. After 24 hours, cells were washed and grown in the absence of serum and with or without 20 µg/ml recombinant human TGFBI (Targetome SA, Belgium) or 20 µg/ml of anti-TGFBI antibodies (10G9A10 and 4G6B10 clones, for description see Supplemental Data). CM were collected, filtered and used for the *in vitro* assays. Proliferation assay: HUVECs (15,000/cm^2^) were incubated with the collected CM (100%) in the presence of 2.5% FCS for 24 h. Then, cells were detached and counted with a MACSQuant cytofluorimeter (Milteny Biotec). Sprouting assay: HUVEC spheroid aggregates were embedded in fibrin gel and stimulated with 50% of CM in the presence of 5% FCS. After 24 hours, growing cell sprouts were photographed and counted under an inverted microscope (Carl Zeiss Vision GmbH). Wound healing assay: HUVEC monolayers were scratched with a 200 µL tip to obtain a 2-mm-thick wound and cultured in the presence of 100% CM with 3.5% FCS. After 18 hours, cell monolayers were photographed and wound healing (percentage of covered area) was quantified with the Fiji software 21.

### *In vivo* CRC models

For the chick embryo chorioallantoic membrane (CAM) *in vivo* CRC model 22, on embryonic day 11, 2×10^6^ SW1222 CRC cells suspended 1:1 in culture medium with Matrigel (BD Biosciences, Bedford, MA) (100 µl final volume) were inoculated on CAMs. Tumor volume was estimated assuming the ellipsoid shape and using the formula V= 4/3.π.((l.w.h)/8), where l, w, h represent the tumor length, width and height, respectively.

For the orthotopic model of CRC liver metastasis formation, SW1222, HT29 and HCT116 cells were injected in the spleen of NOD-SCID mice (Janvier Labs, Saint Berthevin Cedex, France). All experimental procedures for this study were performed in accordance with the ARRIVE ethical guidelines 23, and were approved by the Institutional Animal Care and Ethics Committee of the University of Liège (Belgium). The study adhered to the “Guide for the Care and Use of Laboratory Animals” prepared by the Institute of Laboratory Animal Resources, National Research Council, and published by National Academy Press, as well as to European and local legislation. Mice were anesthetized with 75 mg/kg of ketamine (CEVA, Brussel, BE) and 10 mg/kg of xylazine (Rompun^®^, Bayer, Diegem, BE), and spleen was surgically exposed for injection of 500,000 cells in 100 µL saline solution with 5 mM EDTA. Liver metastasis development was evaluated at week 6 post-injection.

### TGFBI levels measurement by ELISA in human sera

A homemade sandwich ELISA was used to quantify TGFBI levels in serum samples. MaxiSorp 96-well microtiter plates (Nunc, GmbH, Germany) were coated with 100 µL of anti-TGFBI antibody (clone 4G9A10; for details see Supplemental Data), at the concentration of 1 µg/mL in carbonate buffer, and incubated at 4 °C overnight. Coated wells were washed three times with PBS/Tween-20 (0.05%) and blocked with 200 µL of 10% FBS/PBS at 37 °C for 2h. After washing three times as before, 100 µL of serum sample (1:20 in PBS) was added to each well. Serial dilutions of human recombinant TGFBI (Targetome SA), from 0 µg/mL to 5 µg/mL, were prepared in PBS to serve as calibration curve. Plates were incubated at 37 °C for 1h. After washing, 100 µL of anti-TGFBI antibody (1:500 in 10% FBS/PBS; clone 4G6B10, detailed in Supplemental Data) was added to each well at 37 °C for 1h, followed, after washing, by incubation with 100 µL of anti-mouse IgG (1:3000 in 10% FBS/PBS; Dako; cat. no. P0260) at 37 °C for 1h. After washing, wells were incubated with 30% H_2_O_2_ and 1 mM 2,2'-Azinobis-[3-ethylbenzothiazoline-6-sulfonic acid] (ABTS) solution, and the optical density was read using a Filter Max F5 plate reader (Molecular Devices, Sunnyvale, CA, USA) at 405 nm.

### ^89^Zr-radiolabeling of monoclonal antibodies against TGFBI

Anti-TGFBI antibodies were radiolabeled using a previously described three-step procedure 24: (1) antibody coupling of the p-isothiocyanatobenzyl-desferrioxamine chelate, (2) chelated antibody radiolabeling with ^89^Zr oxalic acid, and (3) radiolabeled antibody purification by exclusion chromatography on a Sephadex G25 matrix. After radiolabeling yield and volume activity evaluation, thin layer chromatography was performed to check the absence of free ^89^Zr in the radiolabeled antibody solution. Finally, the radiolabeled TGFBI antibody antigen-binding activity was evaluated by ELISA.

### PET/CT imaging of anti-TGFBI antibody bio-distribution

At day 30 after intrasplenic injection of HT29 (TGFBI-positive) or HCT116 (TGFBI-negative) cells, a contrast product (Exitron® nano12000, Miltenyi Biotec, Germany; 50 ul/mouse) was injected in NOD-SCID mice to highlight liver and spleen by X-ray computed tomography (CT). Then, 100 µg of ^89^Zr-radiolabeled 4G6B10 anti-TGFBI antibody was injected intravenously in 10 mice/model (4-5MBq/mouse at the time of injection). Positron emission tomography (PET)/CT imaging was performed using a preclinical nanoScan PET/CT scanner (Mediso, Hungary). Mice were anaesthetized with isoflurane gas for the examination duration (induction: 4l O_2_/min, 3.5% isoflurane; maintenance: 1.5l O_2_/min, 1.75% isoflurane). PET/CT images were acquired at day 2 (D2, 30 min scan), D6 (30 min scan) and D14 (45 min scan) after injection of the radiolabeled antibodies. PET data were recorded in 3-to-1 coincidence mode and normal count rate. PET images were reconstructed with a fully three-dimensional iterative algorithm (TeraTomo from Mediso, with 4 iterations, 6 subsets, normal regularization setting, median filtering period defined from iteration counts, and spike filter) to obtain a voxel size of 0.4 mm (“normal” mode). Each PET scan was followed by a 6min CT scan for anatomical localization, as well as PET image attenuation and scatter correction. CT acquisition parameters were tube voltage of 50 kV, tube current of 520 μA, 300 ms per projection, 480 projections per rotation, 4-to-1 frame binning, and cubic reconstructed voxel size of 251 μm. All PET images were also corrected for random counts, dead time and decay. PET-CT image viewing and quantitative analysis were performed with VivoQuant v2.5 (InVicro, MA, USA).

### Statistical analysis

Unless otherwise indicated, statistical analysis was performed using data from three biological replicates, a two-sided unpaired Student's *t*-test, assuming equal variances, and GraphPad Prism (GraphPad Software, Inc., La Jolla, CA, USA; version 5.01). The *t*-test was used when data followed a normal distribution (Shapiro-Wilk test, threshold 0.05). Immunoblots were quantified by densitometric analysis using the Image J software and normalized using beta actin expression level. For IHC evaluation, box-plots were generated using the Sigma Plot software (Systat Software Inc., Chicago, IL, USA; version 11.0). The number of samples is indicated in the legends to figures. Statistical differences of IHC data were tested with the Mann-Whitney-U-test because these results did not follow the normal distribution (Shapiro-Wilk test, threshold 0.05).

The other methods used in this study are described in Supplementary Materials and Methods.

## Results

### TGFBI is detected only in stromal cells in primary CRC, and in both stromal and cancer cells in CRC liver metastases

**Figure 1:**
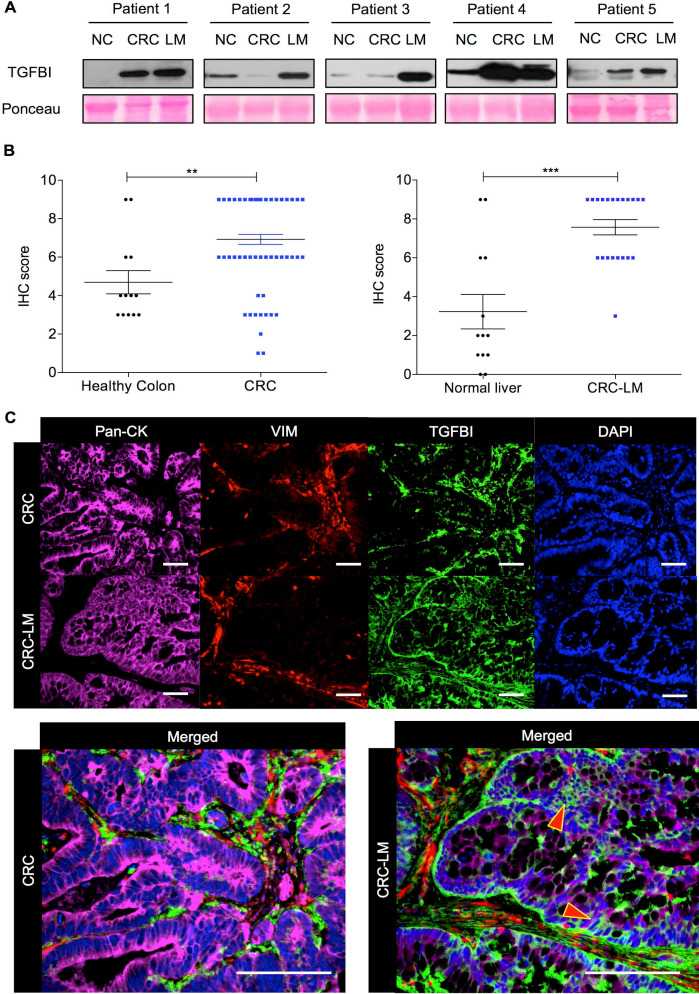
** TGFBI is strongly expressed in CRC and CRC-LM samples. A.** Western blot analysis of TGFBI expression in protein extracts from normal colon (NC), CRC and matched CRC-LM samples from five patients. Ponceau Red staining was used for checking protein loading. **B.** Immunohistochemical analysis of TGFBI expression (IHC score) in CRC (n=78) and CRC-LM (n=21) samples. Adjacent normal colon and normal liver tissues, when available, served as controls (**, *p*<0.01; ***, *p*<0.001; data are the mean ± standard deviation of the mean, SEM). **C.** Immunofluorescence analysis of TGFBI expression in CRC and CRC-LM specimens, co-stained with anti-pan-cytokeratin (Pan-CK; cancer cells), -vimentin (VIM; stromal cells) antibodies and DAPI (nuclei). Representative images of samples from 20 patients.

To precisely determine TGFBI expression in CRC, we initially analyzed by western blotting five pairs of matched CRC/CRC-LM samples, including also normal colon tissue. Regardless of TGFBI expression status in the primary tumor (2/5 CRC samples were nearly negative), TGFBI was always strongly expressed in the matched CRC-LM samples (**Figure 1A**). To understand the source of this expression variability, we then assessed TGFBI expression by IHC in CRC samples from 78 patients and CRC-LM samples from 21 patients (**Figure 1B**). In CRC specimens, TGFBI expression was low (score 2-4) or absent in 12/78 samples, and was medium to high (score 6-9) in the others. In all CRC-LM samples but one, TGFBI expression was high. Adjacent normal colon and liver tissues showed low (limited only to normal stroma) or no staining. Comparison of TGFBI expression by IHC in CRC and CRC-LM samples (representative images in **Figure S1A-B**) indicated that in primary tumors, TGFBI was expressed only by stromal cells. In CRC-LM samples, TGFBI could be detected frequently also in cancer cells (pan-cytokeratin-positive) (**Figure 1C**), although its expression remained predominant in stromal cells (vimentin-positive). Moreover, TGFBI expression in epithelial cancer cells was limited to the cytoplasm/plasma membrane and peri-nuclear space. In stroma, fibroblast cytoplasm was strongly positive as well as the extracellular areas within the zone of desmoplastic reaction. This was in line with literature evidence 15 and our data that TGFBI is a secreted protein (**Figure S1C**). To better understand why only some CRC cells express TGFBI, we next investigated TGFβ signaling in a panel of CRC cell lines and in normal colon fibroblasts.

### TGFβ signaling is active in CRC cell lines that express wild type TGFBR

**Figure 2:**
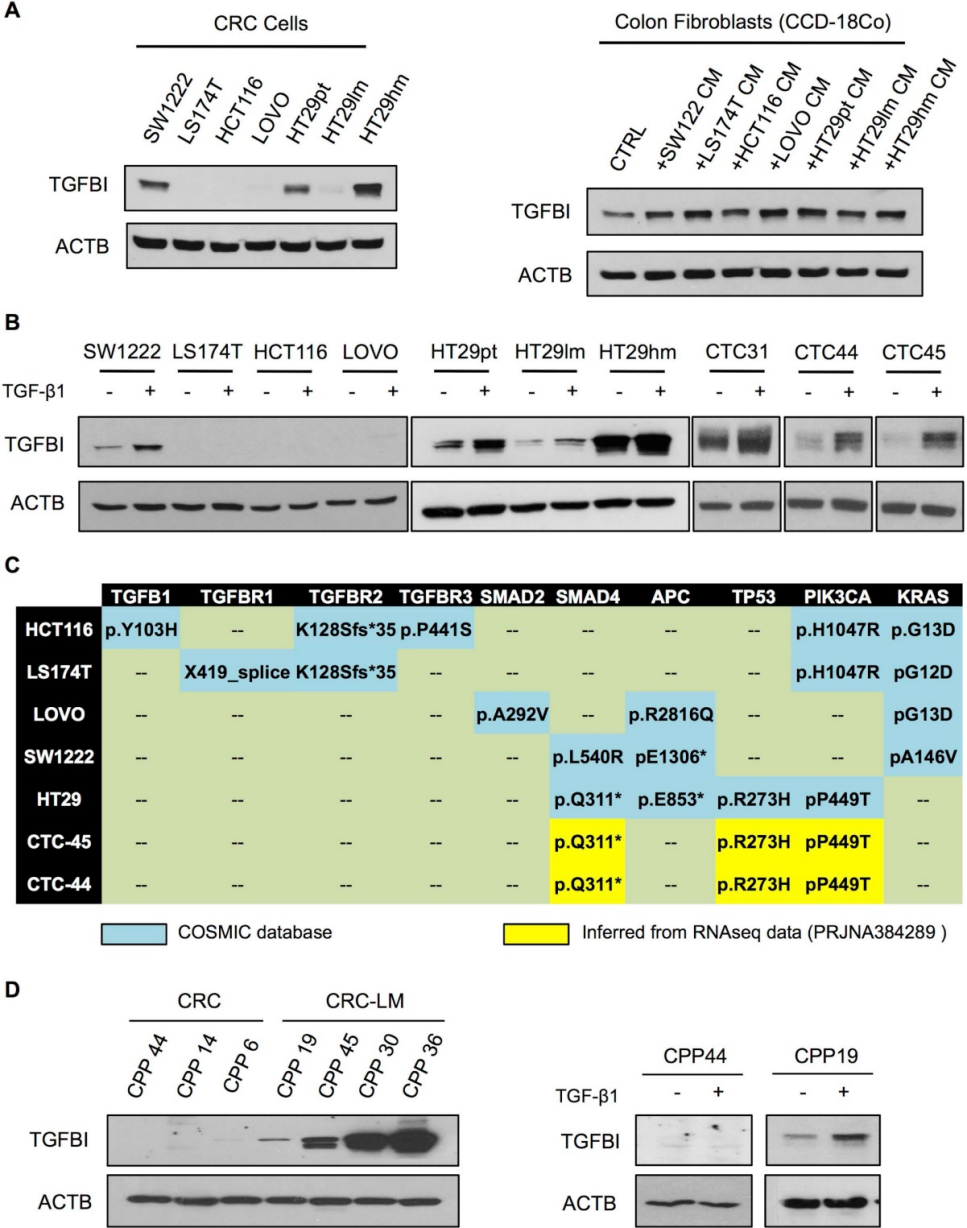
** Metastatic CRC cells maintain TGFβ signaling. A.** Western blot analysis of TGFBI expression in a set of commercially available CRC cell lines (left panel) and in normal colon fibroblasts (CCD-18Co), incubated or not (CTRL) with conditioned medium (CM) from the indicated CRC cell cultures (right panel). Beta actin expression was used as loading control. **B.** Western blot analysis of TGF-β1 responsiveness of CRC cell lines and human CTCs. **C.** Table showing the mutation profile of key TGFβ signaling components and tumor driver genes in CRC and CTC cells (no sequencing information available for CTC31). All cell lines harbored wild type SMAD3 and NRAS. **D.** Basal expression of TGFBI in CPPs isolated from CRC and CRC-LM biopsies (left panel) and after incubation with TGF-β1 (right panel). Panels A, B and D show representative western blot images from three biological replicates.

To investigate TGFβ signaling in CRC cells, we assessed TGFBI basal expression in a panel of commercially available cell lines (**Figure 2A**). Only the HT29 and SW1222 CRC cell lines and CCD-18Co fibroblasts displayed detectable TGFBI levels. Moreover, compared with parental HT29 cells (HT29pt), TGFBI expression was higher in HT29hm cells (high metastatic potential) and lower in HT29lm cells (low metastatic potential) 25. In CCD-18Co fibroblasts, TGFBI expression increased upon incubation with CM from all the tested CRC cell lines. As TGFBI status is indicative of TGFβ signaling, we next asked whether exposure to TGF-β1 could induce TGFBI expression in the tested CRC lines. Only SW1222 and HT29 (all clones) cells were responsive to TGF-β1 exposure (**Figure 2B**). Moreover, analysis of CTCs isolated from the blood of three patients with CRC (CTC31, CTC44, and CTC45) showed that they all expressed TGFBI and were responsive to TGF-β1 exposure. We then investigated the mutation status of the different CRC cell lines (**Figure 2C**). We found frameshift mutations in the TGFβ receptors in HCT116 and LS174T cells that were not responsive to TGF-β1. LOVO cells harbored a SMAD2 mutation. Although SMAD2 is not indispensable for TGFβ signaling, TGFBI expression did not increase in LOVO cells incubated with TGF-β1. Conversely, all tested cell lines in which TGFBI level increased in response to TGF-β1, including CTC44 and CTC45 (for which sequencing data were available; BioProject PRJNA384289), had no mutation in the TGFβ receptors or SMAD2. However, they harbored mutations in SMAD4, which is a mandatory downstream protein in the canonical TGF-β1 pathway. The strongest responders (HT29, CTC44 and CTC45 cells) carried also mutations in p53, which has been described as a mediator of TGFβ signaling together with SMAD2 in human hepatocarcinoma HepG2 cells and mouse embryonic fibroblasts 26. Therefore, we explored the link between TGFβ signaling and p53 mutations in CRC using different approaches. First, we silenced mutant p53 (R273H) in HT29hm cells and tested their response to TGF-β1 (i.e. TGFBI expression modulation), but did not observe any difference between control and p53-silenced HT29hm cells after exposure to TGF-β1 (**Figure S2A-B**). Silencing of wild type p53 (very low protein expression levels before silencing; data not shown) in SW1222 cells did not modify the response to TGF-β1 (**Figure S2C-D**). Conversely, expression of the p53 (R273H) mutant in SW1222 cells led to increased TGFBI expression upon incubation with TGF-β1 (**Figure S2E**). However, this effect was mainly due to increased basal TGFBI expression upon transfection of the p53 mutant in these cells.

To confirm that TGFβ signaling (and hence TGFBI expression) was stronger in cancer cells from metastatic than from primary CRC, we analyzed primary CPPs isolated from CRC and CRC-LM samples. In line with the previous observation, TGFBI was expressed in all CPPs derived from CRC-LM samples, but was not detectable in cells from CRC samples (left panels, **Figure 2D**). Moreover, in CRC-LM-derived CPPs, TGFBI expression could be further increased by incubation with recombinant TGFβ. Conversely, CRC-derived cells did not respond to TGFβ (right panels, **Figure 2D**). Altogether, these results indicated that functional TGFβ receptors are necessary for TGFβ responsiveness, while the contribution of canonical/alternative TGFβ signaling pathways requires more investigations. On the basis of these results, we hypothesized that in CRC, TGFBI levels are maintained through TGFβ signaling in stromal cells, whereas in CRC-LM, TGFBI is also directly secreted by cancer cells that can respond to TGFβ stimulation.

### Alternative TGFβ signaling pathways are responsible for TGFBI induction in CRC cells

**Figure 3:**
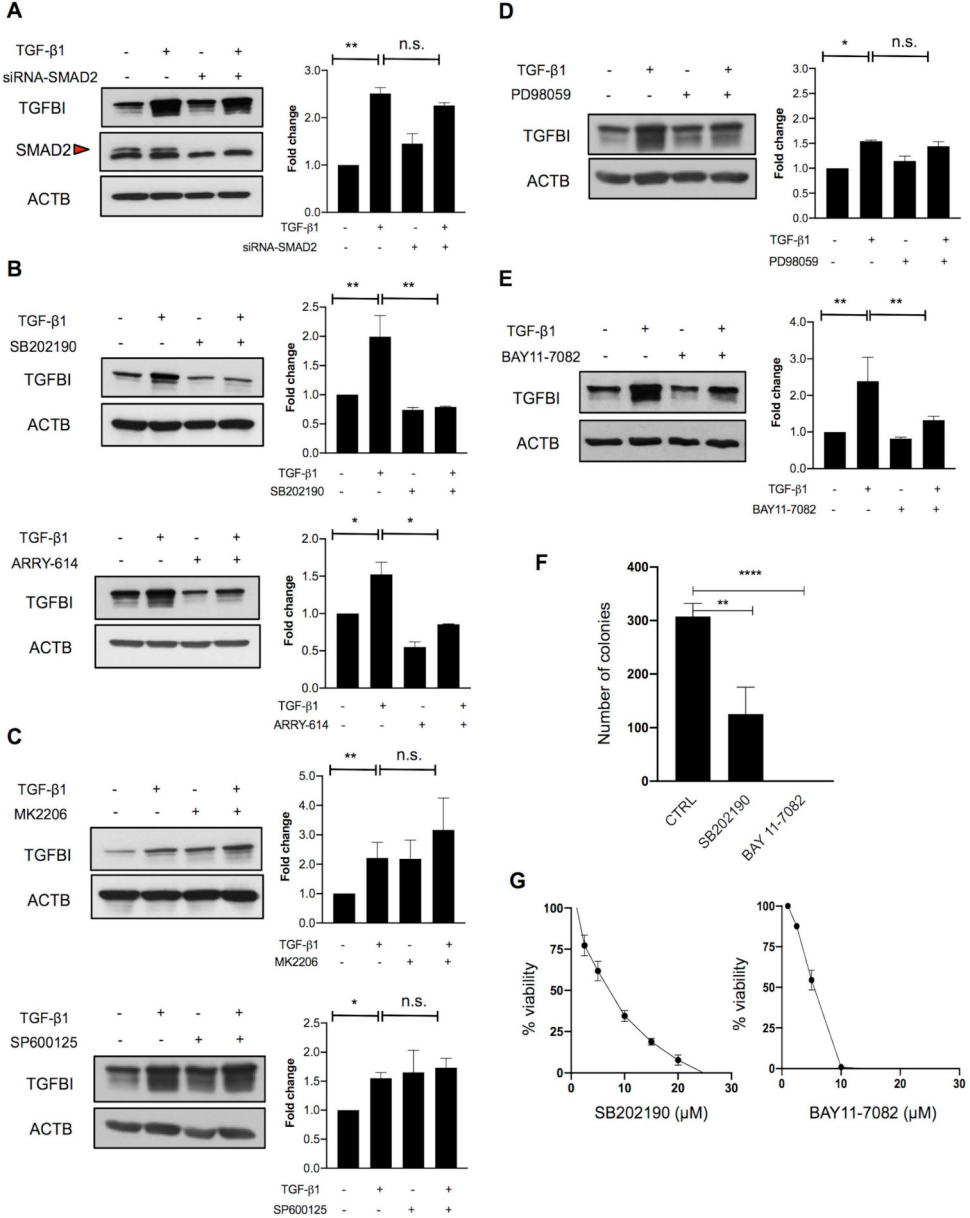
** Alternative TGFβ signaling in CRC is partly driven by p38. A.** Western blot analysis of TGFBI expression in SMAD2-silenced and control HT29hm cells incubated or not with TGF-β1. Beta actin was used as loading control. The involvement of non-canonical pathways was investigated by measuring TGFBI protein levels in HT29hm cells after incubation (for 48h) or not with TGF-β1 (5 ng/ml) or/and the p38 inhibitors SB202190 (5 µM) or ARRY-614 (10 µM) for 48h **(B)**, or/and MK2206 (1 µM, AKT inhibitor), or/and SP600125 (5 µM, JNK inhibitor) **(C)**, or/and PD98059 (5 µM, MAPK inhibitor) **(D)**, or/and BAY11-7082 (5 µM, NFKB inhibitor) **(E).** Panels A-E: representative western blot images from three biological replicates. **F.** Colony formation assay after incubation of HT29hm cells with SB202190 or BAY11-7082 (p38 and NFKB inhibitor, respectively). **G.** HT29hm cell viability analysis after incubation with SB202190 or BAY11-7082. Panels F and G show the mean values ± SEM of biological triplicates. All panels: *, *p*<0.05; **, *p*<0.01; n.s., not significant.

To investigate this hypothesis, we next analyzed the implication of canonical and non-canonical TGFβ signaling. As SMAD2 was not mutated in TGFβ-responsive CRC cells, we first determined whether SMAD2 was required for TGFBI induction. SMAD2 silencing in HT29hm cells did not affect TGF-β1-mediated induction of TGFBI expression (**Figure 3A**). On the basis of the finding that SMAD4 was mutated in all TGFBI-expressing cell lines, we hypothesized that a non-canonical signaling pathway could be implicated in CRC cell response to TGF-β1. Therefore, we tested the implication of p38, AKT, JNK or MAPK in TGF-β1 signaling in HT29hm cells. TGF-β1-mediated induction of TGFBI expression was dampened by incubation of HT29hm cells with selective inhibitors of p38 (SB202190 and ARRY-614) (**Figure 3B**), but not with AKT (MK2206) or JNK (SP600125) inhibitors (**Figure 3C**). Inhibition of MEK1/MEK2 (PD98059) slightly reduced, but not significantly, HT29hm cell response to TGF-β1 (**Figure 3D**). These data suggest that p38 is implicated in TGF-β1-mediated induction of TGFBI expression in HT29hm cells. As p38 cannot function as a transcription factor, we then inhibited NFKB, a transcription factor that can mediate p38 signaling. Incubation with BAY11-7082, a selective NFKB inhibitor, reduced TGFBI expression increase after TGF-β1 stimulation of HT29hm cells (**Figure 3E**). Then, we determined whether p38 and NFKB inhibitors could affect also HT29hm cell growth and viability. These inhibitors significantly reduced the number HT29hm colonies (**Figure 3F**), at least in part by regulating their viability (**Figure 3G**).

Finally, we repeated the same experiments in SW1222 cells, the other TGFβ-responsive cell line. In SW1222 cells, none of the selective MAP kinase inhibitors used in HT29hm cells dampened the response to TGF-β1 stimulation (**Figure S3A-D**). Similarly, NFKB inhibition did not reduce SW1222 cell response to TGF-β1 stimulation (**Figure S3E**). Differently from what observed in HT29hm cells, p38 inhibition in SW1222 cells strongly induced TGFBI expression in basal conditions and also after incubation with TGF-β1 (**Figure S3A**). These findings indicate that the mechanisms underlying TGF-β1 signaling vary in the different CRC cell lines. Therefore, therapeutic strategies should focus more on downstream proteins with effector functions. Next, we sought to better understand TGFBI role in CRC.

### TGFBI silencing reduces tumor growth *in vivo* and suppresses angiogenesis *in vitro*

As previous studies reported that TGFBI can either promote or inhibit progression of different cancer types, it was important to verify TGFBI effect in our CRC models. For this purpose, we determined the impact of TGFBI level modulation on SW1222 cell migration and proliferation. *TGFBI* silencing in SW1222 cells strongly reduced their migration (**Figure S4A-B**), and decreased their viability (MTT assay) and ability to form colonies (**Figure S4C-E**). Accordingly, incubation with TGFBI increased SW1222 cell proliferation and migration capacity (**Figure S4F-G**). To understand the underlying mechanism, we performed a proteomic analysis of SW1222 and HT29 cells and CM (to identify extracellular proteins) after *TGFBI* silencing. Proteins involved in JAK-STAT signaling, activation of RAP1 and RAC1 small GTPases, as well as in detoxification and photolytic-degradation processes were upregulated (**Figure S5** and **Table S4**). Proteins involved in metabolism and infectious diseases were downregulated after *TGFBI* silencing compared with control cells (**Figure S6** and**Table S4**).

**Figure 4:**
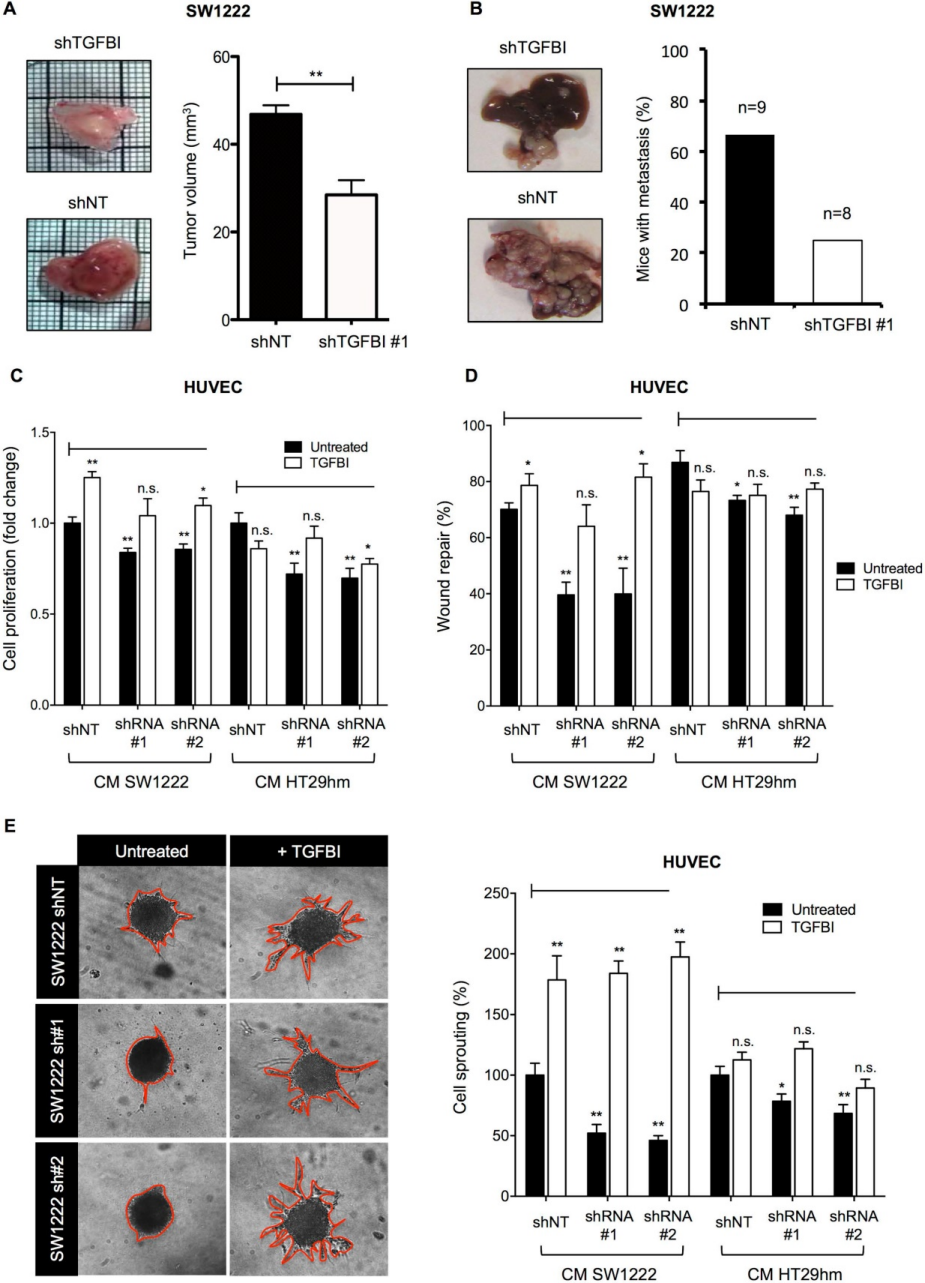
** TGFBI promotes tumor growth and angiogenesis. A.** Tumor growth and angiogenesis in the CAM model using TGFBI-silenced or control (non-target shRNA, shNT) SW1222 cells (left panels) and quantification of tumor volume (right). **B.** Orthotopic liver metastasis model using the same cell lines as in (A). **C.** Proliferation and **D.** Wound healing assays using HUVECs incubated with conditioned media from TGFBI-silenced or control (shNT) SW1222 and HT29 cells and with/without recombinant TGFBI. **E.** HUVEC sprouting assay; same conditions as in panels (C) and (D). Representative images (let panels) and quantification (right). Panels A, C-E: graphs represent the mean ± SEM of quadruplicate biological experiments; *,* p*<0.05; **, *p*<0.01 (vs untreated shNT); n.s., not significant.

Then, we examined TGFBI effects *in vivo* using the CAM tumor model and an orthotopic mouse model of liver metastasis formation. Tumor growth on CAM (**Figure 4A**) and liver metastasis formation in mice (**Figure 4B**) were reduced when *TGFBI*-silenced SW1222 cells were inoculated compared with control SW1222 cells. These *in vivo* experiments also highlighted a markedly lower level of vascularization in tumors derived from *TGFBI*-silenced SW1222 cells, especially in the CAM model. To clarify TGFBI potential role in angiogenesis, we tested the effect of CM from control and *TGFBI*-silenced SW1222 and HT29 cells on HUVECs. CM from *TGFBI*-silenced cancer cells significantly decreased HUVEC proliferation, migration and sprouting (**Figure 4C-E**). However, addition of recombinant TGFBI to the CM from *TGFBI*-silenced cells blocked their inhibitory effect on proliferation and migration and promoted sprouting (**Figure 4E**). Then, to understand the potential mechanism underlying TGFBI pro-angiogenic function, we carried out a proteomic analysis of HUVECs and found that incubation with recombinant TGFBI activated spliceosome- and lysosome-related pathways and decreased processes related to platelet activation, axon guidance and cellular response to stress (**Figures S7 and S8** and **Table S4**). These data indicate a TGFBI tumor-promoting role in CRC and the need to develop strategies to target this protein.

### Monoclonal antibodies against TGFBI suppress angiogenesis *in vitro* and are candidate diagnostic tools *in vivo*

**Figure 5:**
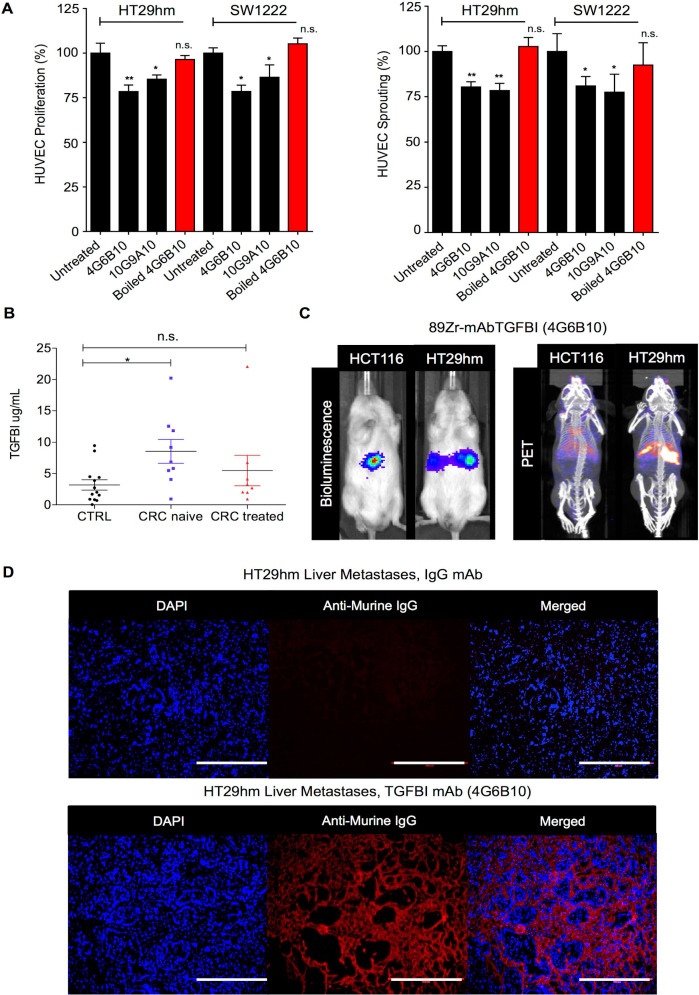
** TGFBI is a CRC-LM diagnostic marker. A.** Targeting HUVEC proliferation (left) and sprouting (right) using anti-TGFBI antibodies *in vitro*. **B.** TGFBI level quantification by ELISA using the 4G6B10 and 10G9A10 anti-TGFBI antibodies in serum samples from healthy controls (CTRL; N=15) and patients with CRC treated (N=8) or not (naïve; N=9) with chemotherapy (for further details see Table S1). **C.** Comparison between bio-luminescence (left) and PET/CT (right) imaging using 89Zr-radiolabeled 4G6B10 (day 6) in mice with liver metastases after intrasplenic injection of the indicated cell lines. **D.** Ex-vivo detection in liver metastases of the 4G6B10 antibody or irrelevant IgG after intravenous injection in the HT29hm orthotopic mouse model (representative images of 5 animals/group). Panels A and B: mean values ± SEM of biological triplicates; *, p<0.05; **, p<0.01; n.s., not significant. Panels C and D: representative images of 4 biological replicates.

To develop novel antibodies against TGFBI, we carried out a *de novo* immunization and screening process that led to the selection of nine anti-TGFBI antibodies that did not cross-react with POSTN (**Figure S9A**). We chose and validated *in vitro* two clones, 10G9A10 and 4G6B10, using FACS, western blotting, surface plasmon resonance (SPR), and immunofluorescence analyses (**Figure S9B-E**). We then tested whether these two anti-TGFBI antibodies could inhibit angiogenesis. To this aim, we monitored HUVEC proliferation and sprouting after incubation with CM from SW1222 and HT29hm cells, in the presence or not of these two antibodies. Both clones significantly inhibited HUVEC proliferation and sprouting (**Figure 5A**), and this effect was abrogated after antibody denaturation. As monoclonal antibodies are currently used also for diagnostic purposes, we developed a sandwich ELISA assay based on the two anti-TGFBI antibodies to screen serum samples from patients with CRC who were or not treated by chemotherapy (patient clinical characteristics in **Table S1**) and from healthy controls. This assay allowed detecting TGFBI in the serum of healthy individuals and patients (**Figure 5B**). However, TGFBI serum levels were significantly higher in untreated patients, whereas levels were comparable in patients under treatment and in healthy controls. Then, we investigated whether the 4G6B10 anti-TGFBI antibody could be also used for *in vivo* imaging of liver metastases. We selected this clone because of its higher target binding affinity (SPR analysis in **Figure S9D**). After labeling with the ^89^Zr PET tracer, we injected the purified antibody in mice bearing HT29hm or HCT116 (negative control because this cell lines does not express TGFBI) liver metastases. We could clearly detect a PET signal in mice with HT29hm liver metastases already at D2 post-injection (data not shown). The signal intensity peaked at D6 (**Figure 5C**), and was still detectable at D14 (data not shown). To further examine the signal specificity and to gain information on its distribution, we injected IgG (control) or the anti-TGFBI antibody in mice bearing HT29hm liver metastases, and recovered livers at D6 post-injection. Histological analysis (**Figure 5D**) showed a very strong accumulation of the anti-TGFBI antibody especially in the stroma of liver metastases, with very little or no staining in HT29hm cells. This suggests that antibodies against TGFBI cannot be internalized.

## Discussion

The TGFβ superfamily includes many different cytokines, such as TGF-β, activin, bone morphogenetic proteins (BMPs) and six receptors. These receptors show variable affinities to ligand groups and signal through canonical (SMAD-mediated) and non-canonical pathways (ATK, MAPK and others). In normal colon mucosa, the BMP/TGFβ gradient is inversely proportional to the epithelial cell differentiation degree, and is important for stem cell maintenance in the colon crypts. Our current understanding of TGFβ role during malignant transformation is strongly limited to its tumor inhibiting and tumor promoting functions. Both are viewed as temporally separated processes, and solely caused by loss-of-function mutations in members of the TGFβ superfamily 7. These events are thought to segregate early from late steps of CRC development, drawing a line between TGFβ tumor inhibiting and promoting roles. For example, SMAD4 mutations predispose patients with juvenile polyposis to gastrointestinal tumors 27. Patients with high microsatellite instability CRC present multiple mutations of TGFβ signaling components and have lower TGFβ activity and longer survival times 28. On the other hand, in epithelial cancer cells, TGFβ exerts its anti-tumor function by downregulating calcium-binding EGF domain-1 (CCBE1), which is essential for tumor lymphangiogenesis 29. However, CRC with the highest TGFβ activity have the worst clinical prognosis 30. As cancer cells can present mutations in several TGFβ signaling components, it is commonly thought that this pathway is not directly active in cancer cells. Therefore, it has been hypothesized (largely on the basis of the results from animal studies) that CAF orchestrate the pro-tumorigenic TGFβ activity in CRC 8. In this model, TGFβ activates SMAD3 in CAF that in turn produce several pro-metastatic factors, such as ANGPTL4, PTHLH, CTGF and JAG1. Therefore, targeting TGFβ should represent a real treatment opportunity, especially during the late phases of CRC development. However, clinical trials with various TGFβ inhibitors have been rather disappointing in patients with different tumor types, including CRC 31. This strongly suggests that TGFβ tumor inhibiting and promoting functions cannot be temporally separated, and that the mechanisms underlying its cell type-specific pro- and anti-tumor functions need to be revisited.

In the present work, we focused on TGFBI, the upregulation of which indicates activation of the TGFβ pathway in different cell models. In colon, *TGFBI* is a marker to distinguish normal mucosa from benign adenoma and colon cancer 32. Increased *TGFBI* mRNA expression positively correlates with the transition from normal colon to cancer. We and others found that TGFBI is significantly upregulated in primary and metastatic CRC 17, 22, 33. Moreover, many evidences implicate TGFBI in tumor progression. However, very little is known about its relationship with TGFβ, besides the fact that TGFβ induces TGFBI expression. Yet, this knowledge is crucial to understand TGFBI role in the early phases of tumor progression. *Tgfbi* knockout mice are predisposed to multiple cancers, including CRC 16, and display also alterations in cartilage and bone formation 34. This suggests that TGFBI basal expression, as observed in normal colon fibroblasts, is required for its homeostatic function. In normal colon, TGFβ produced by resident fibroblasts exerts autocrine and paracrine effects. TGFβ activity in fibroblasts stimulates extracellular matrix production and basement membrane formation, which serves as an important support for epithelial cells, contributing to epithelial polarity 35. TGFBI might be implicated in this process. However, TGFBI role in normal colon physiology remains to be clarified. Here, we found that TGFBI expressed by metastatic CRC cells has a pro-tumorigenic function by affecting the crosstalk between cancer and endothelial cells, resulting in enhanced angiogenesis.

TGFBI role in CRC might be better understood by investigating the upstream signaling that controls its expression. In the last two decades, few studies reported that CRC epithelial cells respond to TGFβ, although these cells often harbor numerous mutations in TGFβ receptors and downstream signaling components 11, 36, 37. Therefore, it is important to determine how TGFβ switches from tumor suppressor to tumor promoter. Interestingly, in other aggressive human tumors, such as melanoma and glioblastoma, TGFβ signaling is maintained in cancer cells, mainly through non-canonical signaling cascades (PI3K/AKT and RAS/MAPK pathways) 38. In these tumors, canonical SMAD-dependent signaling also contributes to TGFβ pro-metastatic functions, particularly by upregulating genes involved in epithelial to mesenchymal transition, such as *SLUG* and *SNAIL* 39, 40. Here, we found that TGFBI signaling follows an alternative, non-canonical pathway that in some CRC cell lines relies on p38. Additionally, mutations in p53 increased the basal levels of TGFBI protein, but did not directly affect the amplitude of the response to TGFβ. Our findings do not exclude the implication of other not yet identified, non-canonical pathways. Finally, our results may help to develop new therapeutic strategies for CRC; however, their implementation will inevitably require a personalized approach. Indeed, we showed here that not all CRC cell lines (and most probably not all patients) use the same alternative TGFβ signaling pathway. In the light of this complexity, targeting the effector proteins of this alternative signaling cascade (for instance, TGFBI) might be a more promising strategy. Our study confirms that TGFBI is certainly one of such targets in CRC, and shows for the first time that *in vivo* TGFBI induces angiogenesis and is accessible to therapeutic antibodies. Future studies should determine how more potent anti-TGFBI antibodies can be generated, and how TGFBI targeting could be combined with other therapies that affect cancer cell proliferation, metabolism or immunity.

## Supplementary Material

Supplementary figures and tables.
