# Supplementary material for: Metastatic colorectal cancer cells maintain the TGFβ program and use TGFBI to fuel angiogenesis
Source: Theranostics. 2021 Jan 1;11(4):1626–40. doi: 10.7150/thno.51507 (PMC7778592; doi:10.7150/thno.51507)
Supplement: Supplementary file 1 — Supplementary figures and tables. [file thnov11p1626s1.pdf]

Supplementary Material

Metastatic colorectal cancer cells maintain the TGFβ program and use TGFBI to fuel angiogenesis

Barbara Chiavarina *[1,2,3,4], Brunella Costanza[5]*, Roberto Ronca[6], Arnaud Blomme[5], Sara Rezzola[6], Paola Chiodelli[6], Ambre Giguelay[1,2,3,4,7], Guillame Belthier[2,4,8,9], Gilles Doumont[10], Gaetan Van Simaeys[10,11], Simon Lacroix[10,11], Takehiko Yokobori[12], Bilguun Erkhem-Ochir[12], Patrick Balaguer[2,3,4,13], Vincent Cavailles[2,3,4,13], Eric Fabbrizio[2,3,4,14], Emmanuel Di Valentin[15], Stephanie Gofflot[16], Olivier Detry[17], Guy Jerusalem[18], Serge Goldman[10,11], Philippe Delvenne[19], Akeila Bellahcène[5], Julie Pannequin[2,4,8,9], Vincent Castronovo[5†] and Andrei Turtoi[1,2,3,4,12†]

[1]Cancer Research Institute of Montpellier, Tumor Microenvironment and Resistance to Treatment Laboratory, INSERM U1194, Montpellier, France.
[2]Institut National de la Santé et de la Recherche Médicale, Montpellier, France.
[3]Institut du Cancer de Montpellier, Montpellier, France.
[4]Université de Montpellier, Montpellier, France.
[5]Metastasis Research Laboratory, GIGA Cancer, University of Liège, Liège, Belgium.
[6]University of Brescia, Department of Molecular and Translational Medicine, Brescia, Italy.
[7]Cancer Research Institute of Montpellier, Cancer Bioinformatics and Systems Biology Team, INSERM U1194, Montpellier, France.
[8]Institut de Génomique Fonctionnelle, Montpellier, France.
[9]Centre National de la Recherche Scientifique, Montpellier, France.
[10]Center for Microscopy and Molecular Imaging (CMMI), Université libre de Bruxelles (ULB), rue Adrienne Bolland 8, B-6041 Charleroi (Gosselies), Belgium.
[11]Nuclear Medicine department, ULB Hôpital Érasme, route de Lennik 808, B-1070 Brussels, Belgium.
[12]Gunma University Initiative for Advanced Research, International Open Laboratory, Universities of Liege and Montpellier Laboratory, Gunma University, Gunma, Japan.
[13]Cancer Research Institute of Montpellier, Hormone Signaling and Cancer Laboratory, Montpellier, France.
[14]Cancer Research Institute of Montpellier, Oncogenic Pathways in Cancer Laboratory, INSERM U1194, Montpellier, France.
[15]GIGA-Viral Vectors Platform, University of Liège, Liège, Belgium.
[16]BIOTHEQUE, University of Liege, Liege, Belgium.
[17]Department of Abdominal Surgery, University Hospital, University of Liège, Liège, Belgium.
[18]Department of Medical Oncology, University Hospital, University of Liège, Liège, Belgium.
[19]Department of Pathology, University Hospital, University of Liège, Liège, Belgium.

Supplemental Material and Methods

Gene expression analysis

After washes in PBS, total RNA was isolated from cell monolayers using the High Pure RNA Isolation Kit (Roche, Mannheim, Germany; cat. no. 11828665001). Following reverse transcription of 1 mg total RNA with the Transcriptor First Strand cDNA Synthesis Kit (Roche; cat. no. 04897030001), cDNA (100 ng) was mixed with primers (0.5 μM), human UPL-probe system (0.2 μM) (Roche; cat. no. 04683633001), and 2X Fast Start Universal Probe Master mix (Roche; cat. no. 04914058001). DNA was amplified using the LightCycler480 system (Roche) and the following conditions: 95 ºC for 10 min, then 40 cycles of 95 ºC (15 sec) and 60 ºC (1 min). The *TGFBI* primer sequences were: forward 5'-cgagtgctgtcctggatatg-3' and reverse 5'-cccagggtctcgtaaaggtt-3' and UPL probe #66. The *P53* primer sequences were: forward 5'- cagccaagtctgtgacttgcacgtac-3' and reverse 5'-ctatgtcgaaaagtgtttctgtcatc-3'. The relative gene expression levels were normalized to the 18S rRNA levels (Life Technologies, Carlsbad, CA, USA; cat. no.: 4310893E). Experiments were performed in triplicate.

Proliferation Assay

One hundred thousand SW1222 cells were seeded in 24-well plates in complete medium and were left to adhere for 24h. Cells were then washed twice in PBS and starved in serum-free DMEM for 16h. Afterwards, cells were incubated with 10 mg/mL of recombinant TGFBI (Targetome) for 24h, 48h, 72h, and 96h. The cell proliferation rate was assessed by measuring the cell DNA content. Briefly, at each time point, cells were sonicated in 1 mL PBS for 10 seconds. 100 μL of this cell suspension was incubated with an equal volume of Hoechst solution (Cat: #382061, Calbiochem) in a 96-well plate under agitation and in the dark for 30 min. The fluorescent signal was read (excitation: 352 nm/emission: 461 nm) using a Filter Max F5 plate reader (Molecular Devices, Sunnyvale, CA, USA).

Migration Assay

Five hundred thousand SW1222 cells were resuspended in serum-free DMEM (containing 0.1% BSA, 1% penicillin/streptomycin) and seeded in the upper part of a Transwell filter (diameter 6.5 mm, pore size 8 μm, Costar, Cambridge, MA) coated with gelatin (100 μg/ml). Human recombinant TGFBI was added at the concentration of 10 μg/mL. The lower part of the Boyden chamber was filled with complete medium (DMEM/10% FBS) as chemoattractant. After incubation at 37 °C for 24h, migrating cells were fixed and stained

with the Diff-Quick Kit (Cat. # 130832. Medion Diagnostics, Düdingen, Switzerland). Representative images for each insert were taken at a 5X magnification and migrating cells were quantified by densitometry using the ImageJ software (National Institute of Health, USA, public access). Three wells (technical replicates) per condition were counted.

Cell viability assay

SW1222 and HT29hm cells were seeded in 96-well plates (5000 cell/well for SW1222 and 3000 cells/well for HT29hm cells) in complete medium and were left to adhere for 24h. Cells were then incubated with SB202190, BAY11-7082, or vehicle for 72h. Cell proliferation was assessed by 3-(4, 5-dimethyl thiazol-2-yl) 2, 5-diphenyl tetrazolium bromide) (MTT) staining (catalog no. M5655, Sigma-Aldrich). Absorbance was measured at 540 nm.

Colony formation assay

For colony formation, HT29 cells were seeded in 6-well plates (500 cells/well) in the presence of absence of SB202190 (10 μM) or BAY11-7082 (5 μM). shNT or shTGFBI SW1222 cells were seeded at a density of 1000 cells/well. After 9 days of culture, cells were washed twice in 1X PBS, fixed, and stained with 0.5% crystal violet in methanol for 30 min. Colonies were counted using the ImageJ software. Experiment were done in triplicate and repeated three times.

Silencing of p53 and introduction of mutant p53 in HT29hm and SW1222 cells

P53 was silenced in HT29hm and SW1222 cells using shRNA-expressing viral particles following the protocol described in Materials and Methods. pLKO-p53-shRNA-941 (shp53), was obtained from Addgene (#25637). Control shRNA was the pLKO.1-Puro (shNT) plasmid (cat#SHC002, Sigma-Aldrich). The pLNCX-Flag-p53-R273H plasmid (kind gift from Dr. Carol L. Prives, Columbia University, New York, USA) was transfected in SW1222 cells using Lipofectamine (Lipofectamine 2000 reagent, catalog no. 11668-019, Life Technologies, Carlsbad, CA, USA).

Proteomic analyses

Protein extracts from cells were prepared as described for western blot analysis (see Materials and Methods). Conditioned media were collected from cancer cells, centrifuged to remove debris, and concentrated using Amicon ultra-filtration devices with 3 kDa cutoff (Merck-Millipore; cat. no. UFC900308). Then, 50 mg of protein extracts or concentrated conditioned media were reduced by adding 20 mM DTT at 60 °C for 30 min, followed by

alkylation using 50 mM 2-chloroacetamide at RT for 30 min. Proteins were precipitated using the 2D Clean-Up Kit (GE Healthcare, Chicago, IL, USA; cat. no. 80648451). Pellets were suspended in 50 µl 100 mM ammonium bicarbonate buffer (pH 8) containing 1 mM calcium chloride, 0.01% ProteaseMAX surfactant (Promega, Madison, WI, USA; cat. no. V2071) and 1 µg trypsin (Promega; cat. no. V5280). Following overnight trypsin digestion at 37 °C, 1/10 of each sample was transferred into a new tube where all samples were mixed in a library. The library sample underwent peptide fractionation using the High pH Reversed-Phase Peptide Fractionation Kit (Thermo Fisher; cat. no. 84868). Eight individual peptide fractions were eluted and desiccated to dryness. Samples were then dissolved in 0.1% TFA and purified using ZipTip (Merck, Darmstadt, Germany; cat. no. C5737). To calibrate the retention times between the LC-MS runs, all samples were spiked at 25 fmol/µL with PepCalMix (Cat.: #5045759, Sciex, Singapore).

The peptide samples were analyzed using the 1D-nano-HPLC-Q-TOF 6600 system (Sciex, Framingham, MA, USA). One microgram of sample was injected in the C18 column (Acclaim® 75 µm x 150 mm, p/n: 162224; Dionex, California, USA). Peptides were resolved with a gradient of 0–40% phase B (90% acetonitrile, 9.9% water and 0.1% formic acid) for 100 min at the flow rate of 0.3 µl/min. Two acquisition modes were used: data-dependent (DDA) for the library, and SWATH for the individual samples. In the DDA mode the setting was as follows: one full scan in the mass range 400 to 1600 m/z, followed by up to 30 MS/MS scans of the most intensive peptides bearing +2 or +3 charges. The acquired data for each fraction of the library sample were merged and used for MS/MS database search with the Protein Pilot software (Sciex). For the SWATH acquisition, the DDA method was adapted using the automated method generator embedded in the Analyst software (Sciex). Proteins were quantified using the SWATH algorithm in the Peak View software and the previously generated protein library. Further data analysis was conducted using R. SWATH data were normalized based on the total protein load, estimated by the sum of all MS intensities reported for all proteins found in a given sample. The mean values of 3 replicates per condition were calculated, and ratios of treated versus control samples were calculated. Proteins that showed changes higher than 2-fold were retained for further analysis.

Network analysis using the STRING software

Protein-protein interaction analysis was performed using the online STRING tools, version 10 (www.string-db.org).

Production and validation of anti-TGFBI antibodies (10G9A10 and 4G6B10)

Custom-made murine monoclonal anti-TGFBI antibodies were produced (Diaclone,

France) by immunizing 5 mice by injection in the footpads of 1 µg/footpad of recombinant TGFBI (Targetome SA, Belgium). Cells were collected from lymph nodes, pooled and fused to generate the myeloma X63/AG.8653 (Diaclone), and distributed in 96-well plates. TGFBI reactivity was screened by ELISA. Briefly, 96-well plates were first coated with goat anti-mouse IgG (Diaclone), saturated in PBS/5% BSA. and then incubated with 10 µl/well of hybridoma supernatants. Following extensive washings, 2 ng of biotinylated TGFBI or biotinylated POSTN was added into each well, followed by incubation at RT for 1h. Finally, plates were washed and streptavidin-HRP (Europa Bioproducts; cat. no. PZCJ30H) was added to each well. The signal was revealed using TMB according to the manufacturer's recommendations (1-Step Ultra TMB-ELISA, Thermo Scientific; cat. no. 34028).

Purified anti-TGFBI antibodies (clones 10G9A10 and 4G6B10) were also evaluated using HEK-293 cells transfected with TGFBI or POSTN (Targetome) and cultured in the presence of brefeldin A (Sigma-B7651) for 5h. Unlabeled isotype controls were from Diaclone. Cells were incubated first with Cytofix/Cytoperm buffer (BD Biosciences; cat. no. 554722) at 4 °C for 20 min, and then with the antibodies under study at different dilutions (1 µg to 0.01 µg/well) in BD Perm/Wash buffer (BD Biosciences; cat. no. 554723) at 4 °C for 30 min. Following washes, the secondary antibody GAM-FITC (MP Biomedicals; cat. no. 55526) (dilution 1/800) was added at 4 °C for 30 min. Cells were analyzed using a Guava® easyCyte flow cytometer (Millipore).

Binding of anti-TGFBI antibodies was evaluated by surface plasmon resonance (SPR) analysis and immunofluorescence. Binding of the antibodies 4G6B10 and 10G9A10 to immobilized TGFBI was assessed using a BIAcore X-100 apparatus (Cytiva, Marlborough, MA, USA). TGFBI (20 µg/ml in 10 mM sodium acetate, pH 4.0) was allowed to react with a flow cell of a CM5 sensor chip previously activated with a mixture of 0.2 M N-ethyl-N'-(3-dimethylaminopropyl)-carbodiimide hydrochloride and 0.05 M N-hydroxysuccinimide (35 µl, flow rate 10 µl/min). After ligand immobilization, matrix neutralization was performed with 1.0 M ethanolamine (pH 8.5) (35 µl, flow rate 10 µl/min) and activated/deactivated dextran was used as reference (control) system. Increasing concentrations of 4G6B10 and 10G9A10 (from 18.75 to 600 nM) were injected over the TGFBI-coated sensor chip and the response was recorded by tracking the SPR intensity change upon binding progression. Injection lasted for 2 min (flow rate 10 µl/min) to allow the association with immobilized TGFBI and was followed by 10 min of dissociation; each run was performed in HBS-EP buffer (Cytiva) and the sensor chip was regenerated with glycine, pH2. The equilibrium (plateau) values of the SPR sensorgrams were used to build the binding isotherms, after normalization. Binding

isotherm points were fitted with the Langmuir equation for monovalent binding to evaluate the mass surface dissociation constant, Kd. The best-fitting procedure was performed with the SigmaPlot 11.0 software package (Systat Software Inc.).

For immunofluorescence analysis, 5 mm sections from fresh frozen human CRC-LM samples were cut using a cryostat (CM3050, Leica) and fixed in cold methanol. Sections were then incubated with 0.5% Triton X-100 in PBS at RT for five minutes, followed by blocking in 5% BSA at RT for 1h and incubation with the anti-TGFBI antibodies (clones 10G9A10 and 4G6B10) (1:500 dilution in 1% BSA) at 4 °C overnight. Following extensive washing, sections were incubated with the secondary goat anti-mouse antibody coupled to Alexa Fluor® 488 (Thermo Fischer; cat. no. A32723),1:2000 dilution, at RT for 1h. Next, sections were washed and incubated with a mix of anti-vimentin Alexa Fluor® 555 conjugated antibody (Cell Signaling; cat. no. 9855) and anti-pan-cytokeratin Alexa Fluor® 647 conjugated antibody (Cell Signaling; cat. no. 4528), both at 1:50 dilution. Following the final washing step, sections were counterstained with DAPI and mounted for viewing as described in Materials and Methods.

Supplementary Figures

Figure Legends

Table S1: Clinical data of the patients whose serum samples were used for TGFBI measurement by ELISA.

Table S2-S3: Clinical data of the patients whose primary CRC (S2) and CRC-LM (S3) samples were used for immunohistochemistry and immunofluorescence analyses.

Table S4: Proteins identified as significantly upregulated (pink) and downregulated (blue) by proteomic analysis of protein lysates and conditioned medium from HT29hm and SW1222 cancer cells (after *TGFBI* silencing vs control, shNT) and HUVECs (after incubation with recombinant TGFBI vs untreated, CTRL).

Figure S1: Immunohistochemistry analysis of TGFBI expression in (**A**) adjacent normal colon and colorectal cancer (CRC; representative images from 78 patients), and (**B**) adjacent normal liver and colorectal cancer liver metastases (CRC-LM; representative images from 21 patients). Panels **A-B**: 100X magnification. (**C**) High magnification images (400X) of TGFBI staining in epithelial cancer cells and tumor stroma in the matched primary CRC and CRC-LM samples. Arrowheads show TGFBI expression within cancer cells.

Figure S2: Role of p53 in modulating TGFBI expression. (**A**) Validation of shRNA-mediated *p53* silencing in HT29hm cells by quantitative RT-PCR. shNT, not targeted shRNA (control). (**B**) Western blot analysis of TGFBI expression in *p53*-silenced and control HT29hm cells. (**C**) Same as in panel **A,** but using SW1222 cells. (**D**) Same as in panel **B**, but using SW1222 cells. (**E**) Western blot analysis of TGFBI expression in SW1222 cells that overexpress mutant p53 (R273H). Panels **B**, **D** and **E**: representative western blot images from three biological replicates; Panels **A** and **C**: histograms show the mean values ± SEM from biological triplicates; **, $p<0.01$.

Figure S3: Effect of MAP kinase inhibition in SW1222 cells. **A**. Western blot analysis of TGFBI expression in SW1222 cells after incubation with TGF-β1 (5 ng/ml) or/and SB202190 (5 μM, p38 inhibitor), (**B**) MK2206 (1 μM, AKT inhibitor), (**C**) SP600125 (5 μM, JNK inhibitor), (**D**) PD98059 (5 μM, MAP kinase inhibitor), (**E**) BAY11-7082 (5 μM, NFKB

inhibitor) for 48h. Panels **A-E**: representative western blot images from three biological replicates; histograms show the mean values ± SEM of biological triplicates; *, p<0.05; **, p<0.01; ns, not significant.

Figure S4: TGFBI promotes cell proliferation and migration *in vitro*. (**A**). Validation of siRNA-mediated *TGFBI* silencing by quantitative RT-PCR at 48h, 72h and 96h post-transfection. (**B**) Migration assay using *TGFBI*-silenced (siTGFBI) and control (siCTRL) SW1222 cells after 24h incubation. Representative images from three biological replicates. (**C**) Validation of shRNA-mediated *TGFBI* silencing in SW1222 cells by quantitative RT-PCR. (**D**) Viability and (**E**) Colony formation assays using *TGFBI*-silenced (shTGFBI) and control (shNT) SW1222 cells. (**F**) Proliferation assay using SW1222 cells grown with recombinant TGFBI (10 mg/mL) for 24h, 48h, 72h and 96h. (**G**). Migration assay of SW1222 cells incubated with recombinant TGFBI for 24h. Panels **A-G**: histograms show the mean values ± SEM of biological triplicates; *, p<0.05; **, p<0.01.

Figure S5: Network of upregulated proteins in protein lysates and conditioned media of *TGFBI*-silenced SW1222 and HT29hm cells. Significantly enriched pathways are listed in the table.

Figure S6: Network of downregulated proteins in protein lysates and conditioned media of *TGFBI*-silenced SW1222 and HT29hm cells. Significantly enriched pathways are listed in the table.

Figure S7: Network of upregulated proteins found in protein lysates of HUVECs incubated with recombinant TGFBI. Significantly enriched pathways are listed in the table.

Figure S8: Network of downregulated proteins in protein lysates of HUVECs incubated with recombinant TGFBI. Significantly enriched pathways are listed in the table.

Figure S9: Selection and validation of monoclonal anti-TGFBI antibodies. **(A)** ELISA-based screening of nine anti-TGFBI antibodies: binding to recombinant TGFBI (left) or POSTN (right). **(B)** FACS analysis of the two top binding anti-TGFBI antibodies, 10G9A10 and 4G6B10, in HEK-293 cells transfected with TGFBI or POSTN. **(C)** Western blot analysis of TGFBI expression using the 10G9A10 and 4G6B10 antibodies in the indicated CRC cell lines, CRC liver metastasis sample, and CCD18-Co fibroblasts. (**D)** SPR analysis of the

10G9A10 and 4G6B10 antibody target affinity. **(E)** Immunofluorescence analysis of 10G9A10 and 4G6B10 localization in fresh frozen human CRC-LM samples, with vimentin (stromal cell marker) and pan-cytokeratin (cancer cell marker) labeling (representative images of 10 samples).

Patient No.	Age	Pathology	Localization	Treatment Status	TNM	MS status
1	81	adenocarcinoma	Right colon	Naive	pT3N0Mx	MSI
2	60	adenocarcinoma	Right colon	Naive	pT3N0Mx	MSS
3	69	adenocarcinoma	Rectum	Naive	pT3NxMx	NR
4	84	adenocarcinoma	Right colon	Naive	pT4bN0Mx	NR
5	85	adenocarcinoma	Sigmoide	Naive	pT4aN1aM1b	NR
6	75	adenocarcinoma	Sigmoide	Naive	pT4bN1bM1a	NR
7	90	adenocarcinoma	Rectum	Naive	pT3N0M1	NR
8	76	adenocarcinoma	Rectum	Naive	pT3N1M0	NR
9	73	adenocarcinoma	Right colon	Naive	pT4aN0M1a	MSI
10	80	adenocarcinoma	Rectum	Under treatment	pT3NxM1	MSS
11	76	adenocarcinoma	Sigmoide	Under treatment	pT3N2aMx	MSS
12	67	adenocarcinoma	Right colon	Under treatment	pT4aN0Mx	MSS
13	80	adenocarcinoma	Rectum	Under treatment	pT4bN0Mx	MSS
14	71	adenocarcinoma	Sigmoide	Under treatment	pT4aN0Mx	MSI
15	76	adenocarcinoma	Rectum	Under treatment	pT4N3M0	NR
16	33	adenocarcinoma	Left colon	Under treatment	pT4bN1bM1a	MSS
17	52	adenocarcinoma	Sigmoide	Under treatment	pT4aN2aM+	MSS
18	64	Healthy CTRL	NA	NA	NA	NA
19	58	Healthy CTRL	NA	NA	NA	NA
20	63	Healthy CTRL	NA	NA	NA	NA
21	78	Healthy CTRL	NA	NA	NA	NA
22	60	Healthy CTRL	NA	NA	NA	NA
23	63	Healthy CTRL	NA	NA	NA	NA
24	43	Healthy CTRL	NA	NA	NA	NA
25	66	Healthy CTRL	NA	NA	NA	NA
26	70	Healthy CTRL	NA	NA	NA	NA
27	68	Healthy CTRL	NA	NA	NA	NA
28	65	Healthy CTRL	NA	NA	NA	NA
29	52	Healthy CTRL	NA	NA	NA	NA
30	68	Healthy CTRL	NA	NA	NA	NA
31	67	Healthy CTRL	NA	NA	NA	NA
32	65	Healthy CTRL	NA	NA	NA	NA

MSS: microsatellite stable
MSI: microsatellite instable

Table S1

Patient number	Age	pTNM	Tumor Grade	Therapy	Metastases
1	66	pT1N0MX	2	NA	no
2	76	pT1 NX MX	2	NA	no
3	51	pT1N0MX	1	no	no
4	85	pT1N0Mx	2 - 3	no	no
5	61	pT1N0Mx	2 - 3	no	no
6	66	pT1N0Mx	1	chemo+radio	yes
7	78	pT1N1Mx	1	no	no
8	60	p T1N0Mx.	2	no	no
9	48	pT1N0Mx	2	no	no
10	70	pT1N0Mx	2	no	no
11	77	pT1N2bM1b	2 - 3	no	yes
12	79	pT1(SM3) Nx(0/7) Mx	2	no	no
13	74	pT1N0Mx	1	no	NA
14	83	pT1N0MX	2	NA	NA
15	67	pT1N0Mx	2	no	yes
16	75	pT1N0Mx	1-2	no	yes
17	64	pT1sm3N0Mx.	1 - 2	no	no
18	71	pT1 sm1N0Mx	1	no	yes
19	88	pT1 No Mx	1-2	NA	no
20	60	pT1N0Mx	2	NA	yes
21	77	pT2N0Mx	2	no	no
22	68	pT2N0Mx	1	no	no
23	67	pT2N0MX	2	no	NA
24	77	pT2 N0 MX.	1	no	NA
25	64	pT2N0Mx	2	no	no
26	71	pT2N1MX	2	chemo	no
27	53	pT2N1bMx.	2	chemo	no
28	64	pT2N1Mx	2	chemo	no
29	73	pT2(sm1)NxMx	2	no	no
30	79	T2NxMx	3	no	no
31	88	pT2N1b.	1	no	no
32	89	pT2N0Mx	2	no	no
33	83	pT2N0Mx	2	no	no
34	77	pT2N0MX	2	chemo	yes
35	74	pT2NxMx.	2	no	no
36	77	pT2N0(0/8)Mx	2	NA	NA
37	56	pT2 Nx (0/9) Mx.	2	no	no
38	48	pT2 Nx (0/8) Mx	2	no	no
39	67	pT2N0Mx	1	no	no
40	72	pT2N0Mx	2	no	yes
41	67	pT3N1bMx	2	chemo	no
42	77	pT3 Nx(0/10) Mx	2	no	no
43	74	pT3 N0 Mx	2-3	no	no
44	77	pT3 N1c Mx R0	2	chemo	no
45	90	pT3N1aMx	2	NA	NA
46	92	pT3N0Mx	2	no	no
47	57	pT3N2aMx	2	chemo	no
48	76	pT3N2aMx	2	chemo	yes
49	77	pT3N1aMx	2	chemo	no
50	87	pT3 Nx (0/6) Mx	1-2	no	no
51	70	pT3N1aM1a	3	chemo	yes
52	73	pT3N2bMx	2	chemo	no
53	69	pT3N1aMx	2-3	radio	no
54	59	pT3N2aMx	2-3	chemo	no
55	85	pT3N0Mx	2	no	no
56	54	pT3N2aMx	2	chemo	yes
57	77	pT3N0Mx	2	no	NA
58	54	pT3 N2b Mx	2	chemo	yes
59	74	pT3N0Mx	2	no	no
60	75	pT3N0Mx	1-2	no	no
61	68	pT3 N1a Mx	2	chemo	no
62	66	pT4N0Mx	2-3	no	no
63	69	pT4N0Mx.	2-3	chemo	no
64	34	ypT4bN1bM1a.	2-3	chemo	yes
65	90	pT4bN1bMX	2-3	no	no
66	71	pT4aN0Mx	2	chemo	yes
67	67	pT4b N2a Mx.	2	chemo	no
68	78	pT4a Nx (0/6) Mx	2	no	no
69	70	pT4bN0Mx	3	chemo	yes
70	84	pT4bN2bMx	2	no	yes
71	76	pT4aN0Mx	2	no	no
72	83	p T4a N2b Mx	3	no	yes
73	74	pT4a N2a Mx	2	chemo	yes
74	25	pT4b N1a Mx	3	chemo	no
75	57	pT4aN1cM0	2	chemo	yes
76	71	pT4aN1bMx	2-3	chemo	no
77	86	pT4aN1aMx	2	chemo	no
78	84	pT4aN0Mx	2	NA	NA

Table S2

Patient number	Age	Primary Tumor Type	KRAS Status
1	59	poorly differentiated	WT
2	63	poorly differentiated	
3	71	poorly differentiated	
4	52	poorly to moderately differentiated	
5	69		
6	61	mocinous poorly differentiated	
7	53	poorly differentiated	
8	69	moderately differentiated	Mutation codon 12
9	69		
10	75	poorly differentiated	
11			
12		poorly differentiated partially mucoid	Mutation codon 13
13	64		Mutation codon 12
14			
15			
16			
17			
18			
19			
20		poorly to moderately differentiated	Mutation codon 12
21			Mutation codon 12

Table S3

UPREGULATED shTGFBI/shNT							
Cancer Cells				Conditioned Media			
**SprotID**	**Gene Name**	**FC Sw1222**	**FC HT29hm**	**SprotID**	**Gene Name**	**FC Sw1222**	**FC HT29hm**
P00995	SPINK1	19.04	4.84	O00592	PODXL	12.57	57.38
P04424	ASL	2.01	2.12	O15031	PLXNB2	11.91	1215.17
P10586	PTPRF	3.96	2.09	O95865	DDAH2	5.16	14.11
P62633	CNBP	2.28	6.58	P00338	LDHA	4.01	15.45
Q3ZCQ8	TIMM50	2.52	2.27	P01033	TIMP1	3.78	10.63
Q92522	H1FX	2.90	2.21	P03973	SLPI	3.03	5.32
Q92597	NDRG1	2.35	3.37	P06396	GSN	4.43	12.81
Q969S3	ZNF622	2.02	2.44	P08294	SOD3	3.08	32.25
Q9BVI4	NOC4L	7.00	6.02	P10768	ESD	3.95	7.33
Q9NNW7	TXNRD2	4.04	2.44	P13645	KRT10	4.30	98.43
Q9Y6R7	FCGBP	3.92	4.32	P23528	CFL1	4.08	6.94
				P52907	CAPZA1	3.27	5.25
				P60981	DSTN	3.12	3.16
				P61224	RAP1B	5.16	3.98
				P61956	SUMO2	3.43	12.69
				P63000	RAC1	3.87	6.68
				P63165	SUMO1	3.66	4.23
				Q06830	PRDX1	4.21	59.79
				Q14103	HNRNPD	4.13	3.18
				Q96DG6	CMBL	9.71	9.66
				Q9GZU8	FAM192A	3.48	5.47
				Q9HC38	GLOD4	3.90	11.37
				Q9NP84	TNFRSF12A	4.07	4.84
				Q9Y3B8	REXO2	7.81	4.98

DOWNREGULATED shTGFBI/shNT							
Cancer Cells				Conditioned Media			
**SprotID**	**Gene Name**	**FC Sw1222**	**FC HT29hm**	**SprotID**	**Gene Name**	**FC Sw1222**	**FC HT29hm**
O43823	AKAP8	0.17	0.09	O00170	AIP	0.19	0.13
O75487	GPC4	0.28	0.32	O15212	PFDN6	0.11	0.12
O75608	LYPLA1	0.03	0.21	O15498	YKT6	0.12	0.12
O95777	LSM8	0.15	0.32	O43240	KLK10	0.03	0.17
P00966	ASS1	0.36	0.14	O43447	PPIH	0.13	0.19
P02749	APOH	0.38	0.46	O75251	NDUFS7	0.07	0.15
P04114	APOB	0.35	0.16	O75351	VPS4B	0.03	0.12
P07919	UQCRH	0.02	0.07	O75475	PSIP1	0.05	0.11
P09382	LGALS1	0.03	0.10	O75489	NDUFS3	0.09	0.14
P09972	ALDOC	0.25	0.21	O75964	ATP5MG	0.16	0.13
P0C0L5	C4B	0.38	0.42	O76094	SRP72	0.17	0.08
P14324	FDPS	0.34	0.46	O94905	ERLIN2	0.13	0.05
P15289	ARSA	0.06	0.10	P01037	CST1	0.04	0.03
P17900	GM2A	0.08	0.34	P07305	H1F0	0.11	0.13
P19388	POLR2E	0.17	0.15	P07711	CTSL	0.02	0.19
P22676	CALB2	0.34	0.41	P10145	CXCL8	0.10	0.04
P31431	SDC4	0.10	0.49	P10451	SPP1	0.18	0.06
P33316	DUT	0.14	0.26	P13473	LAMP2	0.11	0.09
P34059	GALNS	0.49	0.45	P19623	SRM	0.04	0.15
P52434	POLR2H	0.15	0.42	P20742	PZP	0.17	0.15

Table S4

DOWNREGULATED shTGFBI/shNT							
Cancer Cells				Conditioned Media			
SprotID	Gene Name	FC Sw1222	FC HT29hm	SprotID	Gene Name	FC Sw1222	FC HT29hm
P55010	EIF5	0.26	0.44	P30419	NMT1	0.06	0.14
P62837	UBE2D2	0.42	0.32	P32969	RPL9	0.10	0.18
P84243	H3F3A	0.02	0.27	P36543	ATP6V1E1	0.19	0.16
Q13131	PRKAA1	0.03	0.29	P38571	LIPA	0.02	0.19
Q13885	TUBB2A	0.23	0.04	P46776	RPL27A	0.03	0.11
Q14566	MCM6	0.46	0.49	P47755	CAPZA2	0.18	0.13
Q147X3	NAA30	0.19	0.29	P47985	UQCRFS1	0.17	0.11
Q15582	***TGFBI***	***0.36***	***0.29***	P48449	LSS	0.12	0.14
Q15631	TSN	0.37	0.10	P48556	PSMD8	0.15	0.13
Q15819	UBE2V2	0.14	0.50	P49368	CCT3	0.02	0.18
Q16663	CCL15	0.42	0.21	P53680	AP2S1	0.19	0.11
Q16790	CA9	0.35	0.20	P61106	RAB14	0.17	0.16
Q6P1N0	CC2D1A	0.04	0.02	P62266	RPS23	0.02	0.14
Q6UWK7	GPR15L	0.02	0.21	P62273	RPS29	0.10	0.20
Q7L5L3	GDPD3	0.12	0.09	P62308	SNRPG	0.13	0.15
Q86UD1	OAF	0.30	0.16	P62750	RPL23A	0.03	0.02
Q8NDH3	NPEPL1	0.16	0.06	P62841	RPS15	0.12	0.13
Q8NFU3	TSTD1	0.32	0.03	P68402	PAFAH1B2	0.16	0.10
Q96G03	PGM2	0.01	0.08	P98194	ATP2C1	0.12	0.07
Q96GA3	LTV1	0.45	0.27	Q00765	REEP5	0.07	0.13
Q99674	CGREF1	0.44	0.16	Q03001	DST	0.02	0.18
Q99988	GDF15	0.37	0.15	Q08379	GOLGA2	0.15	0.12
Q9GZT8	NIF3L1	0.49	0.42	Q14061	COX17	0.11	0.17
Q9NR28	DIABLO	0.14	0.05	Q14657	LAGE3	0.01	0.04
Q9UM00	TMCO1	0.20	0.15	Q15006	EMC2	0.07	0.05
				Q15382	RHEB	0.13	0.10
				Q15582	***TGFBI***	***0.11***	***0.05***
				Q15773	MLF2	0.13	0.13
				Q3ZCQ8	TIMM50	0.03	0.18
				Q66K14	TBC1D9B	0.11	0.11
				Q7L5L3	GDPD3	0.18	0.12
				Q7Z2W4	ZC3HAV1	0.07	0.08
				Q86Y46	KRT73	0.00	0.08
				Q8NBJ7	SUMF2	0.07	0.07
				Q8WUJ3	CEMIP	0.08	0.17
				Q8WVN6	SECTM1	0.01	0.01
				Q92878	RAD50	0.12	0.11
				Q96ER9	CCDC51	0.04	0.03
				Q99747	NAPG	0.05	0.07
				Q9BPX5	ARPC5L	0.00	0.08
				Q9BXW7	HDHD5	0.11	0.08
				Q9H223	EHD4	0.18	0.15
				Q9H8S9	MOB1A	0.12	0.04
				Q9NRX4	PHPT1	0.04	0.13
				Q9NX24	NHP2	0.10	0.06
				Q9UBM7	DHCR7	0.16	0.08
				Q9UGI8	TES	0.07	0.06
				Q9UK23	NAGPA	0.12	0.09
				Q9UKM9	RALY	0.18	0.08
				Q9UL25	RAB21	0.18	0.09

Table S4 (continued)

UPREGULATED +TGFBI/CTRL					
SprotID	Gene Name	FC HUVEC	SprotID	Gene Name	FC HUVEC
P15121	AKR1B1	104.37	P14174	MIF	26.28
O14617	AP3D1	73.53	Q9UM54	MYO6	34.02
P52566	ARHGDIB	76.43	P15531	NME1	33.79
P59998	ARPC4	298.27	Q9BPZ3	PAIP2	1091.67
Q12797	ASPH	898.27	P52434	POLR2H	862.35
Q9NVI7	ATAD3A	111.94	P63151	PPP2R2A	37.50
Q08257	CRYZ	120.76	P42785	PRCP	21.19
P04080	CSTB	35.75	P07602	PSAP	845.20
P07339	CTSD	22.43	P25789	PSMA4	20.04
P63172	DYNLT1	5912.78	P20962	PTMS	33.56
Q15006	EMC2	20.42	Q9P0K7	RAI14	121.14
O95571	ETHE1	35.37	Q9Y5S9	RBM8A	79.27
Q01844	EWSR1	22.60	P62244	RPS15A	159.34
Q9UN86	G3BP2	46.04	P62851	RPS25	117.98
P11413	G6PD	50.90	Q9Y230	RUVBL2	27.71
Q04760	GLO1	89.29	Q15424	SAFB	55.67
P15170	GSPT1	34.97	P08621	SNRNP70	677.97
O75367	H2AFY	26.48	P08579	SNRPB2	36.46
Q7Z4V5	HDGFL2	43.69	P62318	SNRPD3	46.35
P52926	HMGA2	94.14	P53999	SUB1	104.46
P26583	HMGB2	25.67	Q8NBK3	SUMF1	39.29
O43390	HNRNPR	79.41	Q14258	TRIM25	29.79
Q96P70	IPO9	307.24	Q9H4B7	TUBB1	27.50
Q12907	LMAN2	102.87	O43396	TXNL1	38.47
P62310	LSM3	27.21	Q9P0L0	VAPA	55.71
			P04004	VTN	21524.41

DOWNREGULATED +TGFBI/CTRL		
SprotID	Gene Name	FC HUVEC
P20073	ANXA7	0.40
P21810	BGN	0.39
Q9Y6A4	CFAP20	0.25
P23528	CFL1	0.40
P61073	CXCR4	0.33
Q13011	ECH1	0.27
O60869	EDF1	0.39
Q9NY12	GAR1	0.25
Q9UBI6	GNG12	0.32
O60814	HIST1H2BK	0.30
Q86YZ3	HRNR	0.27
Q8TF66	LRRC15	0.33
P08253	MMP2	0.31
Q8NCN5	PDPR	0.33
Q15185	PTGES3	0.27
Q09028	RBBP4	0.33
Q02878	RPL6	0.31
Q14108	SCARB2	0.37
P00441	SOD1	0.34
O15260	SURF4	0.25
P68366	TUBA4A	0.38
Q92900	UPF1	0.38

Table S4 (continued)

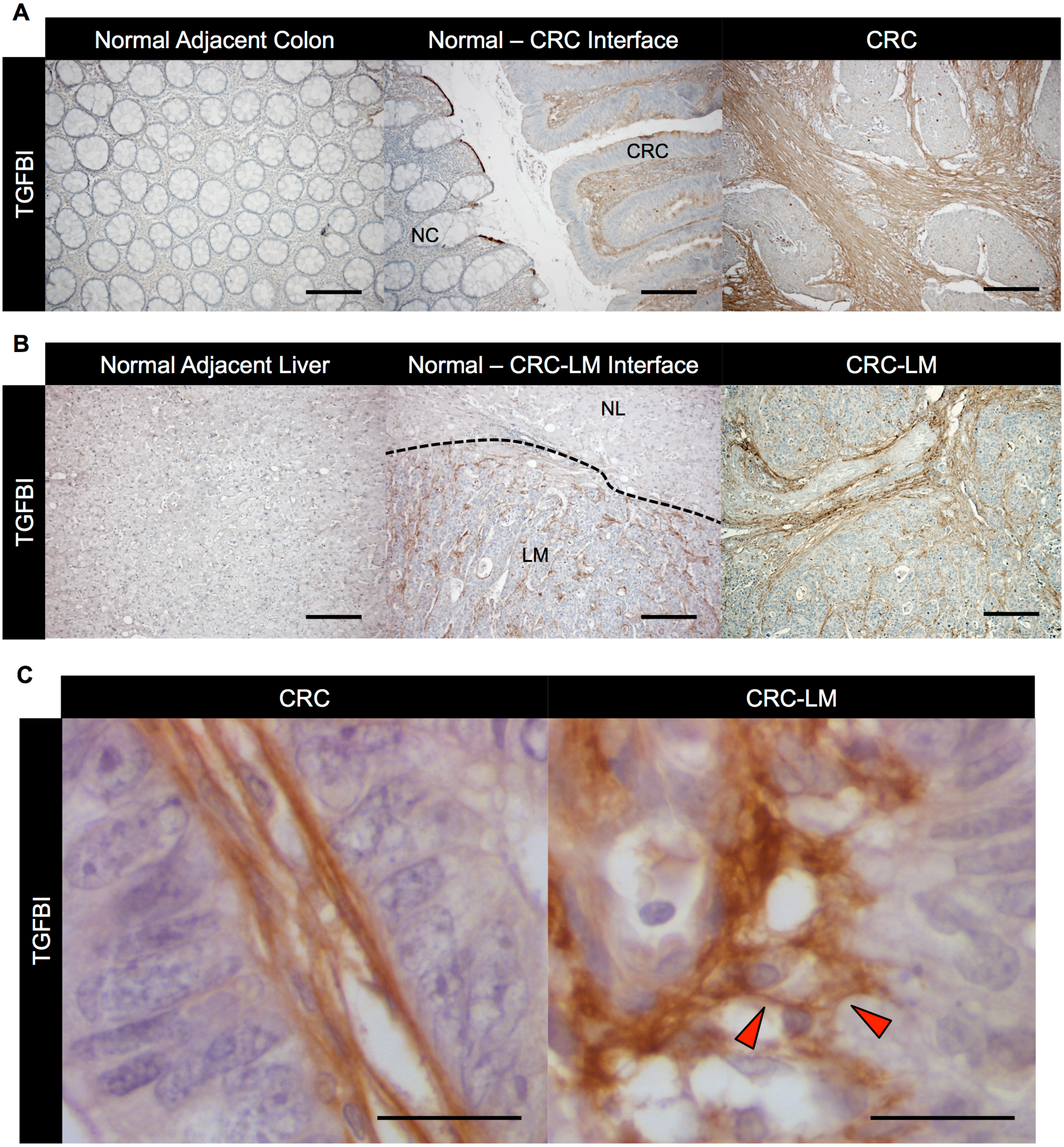
A
Normal Adjacent Colon
Normal – CRC Interface
CRC
TGFBI
CRC
NC
B
Normal Adjacent Liver
Normal – CRC-LM Interface
CRC-LM
TGFBI
NL
LM
C
CRC
CRC-LM
TGFBI

Figure S1

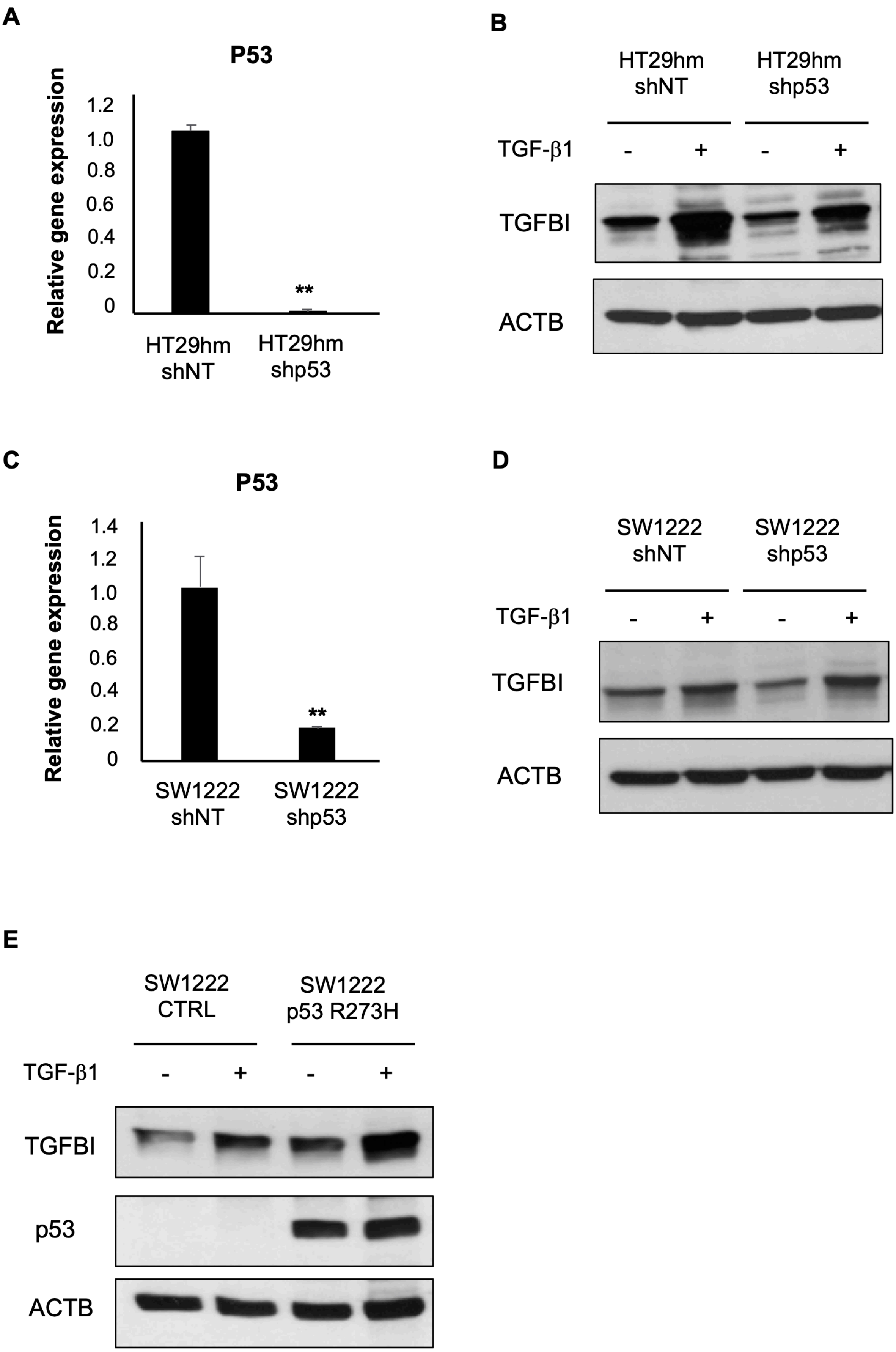
A
P53
Relative gene expression
1.2
1.0
0.8
0.6
0.4
0.2
0
**
HT29hm shNT
HT29hm shp53
B
HT29hm shNT
HT29hm shp53
TGF-β1
-
+
-
+
TGFBI
ACTB
C
P53
Relative gene expression
1.4
1.2
1.0
0.8
0.6
0.4
0.2
0
**
SW1222 shNT
SW1222 shp53
D
SW1222 shNT
SW1222 shp53
TGF-β1
-
+
-
+
TGFBI
ACTB
E
SW1222 CTRL
SW1222 p53 R273H
TGF-β1
-
+
-
+
TGFBI
p53
ACTB

Figure S2

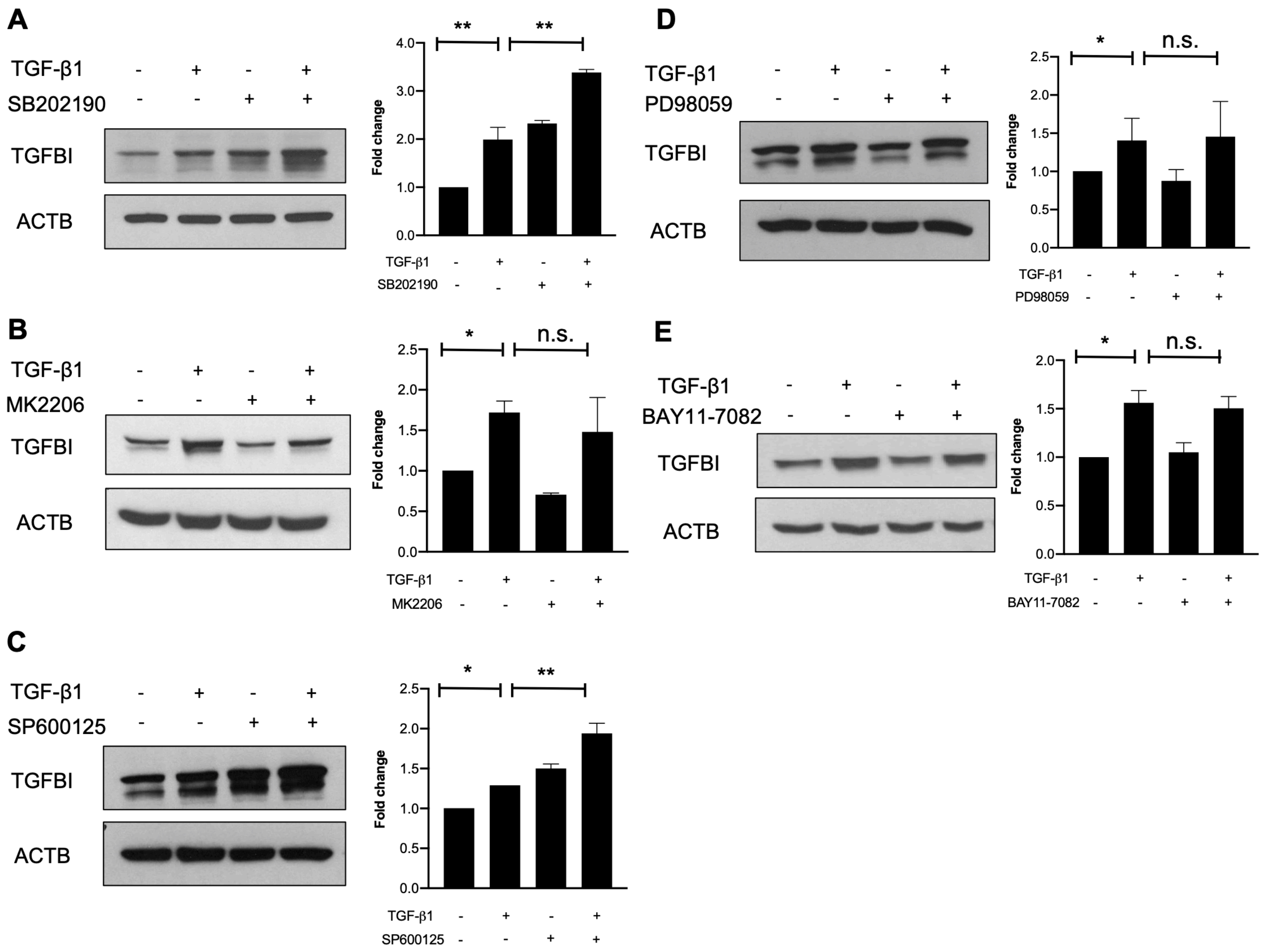
A
TGF-β1 - + - +
SB202190 - - + +
TGFBI
ACTB
**
**
Fold change
4.0
3.0
2.0
1.0
0.0
TGF-β1 - + - +
SB202190 - - + +
B
TGF-β1 - + - +
MK2206 - - + +
TGFBI
ACTB
*
n.s.
Fold change
2.5
2.0
1.5
1.0
0.5
0.0
TGF-β1 - + - +
MK2206 - - + +
C
TGF-β1 - + - +
SP600125 - - + +
TGFBI
ACTB
*
**
Fold change
2.5
2.0
1.5
1.0
0.5
0.0
TGF-β1 - + - +
SP600125 - - + +
D
TGF-β1 - + - +
PD98059 - - + +
TGFBI
ACTB
*
n.s.
Fold change
2.5
2.0
1.5
1.0
0.5
0.0
TGF-β1 - + - +
PD98059 - - + +
E
TGF-β1 - + - +
BAY11-7082 - - + +
TGFBI
ACTB
*
n.s.
Fold change
2.0
1.5
1.0
0.5
0.0
TGF-β1 - + - +
BAY11-7082 - - + +

Figure S3

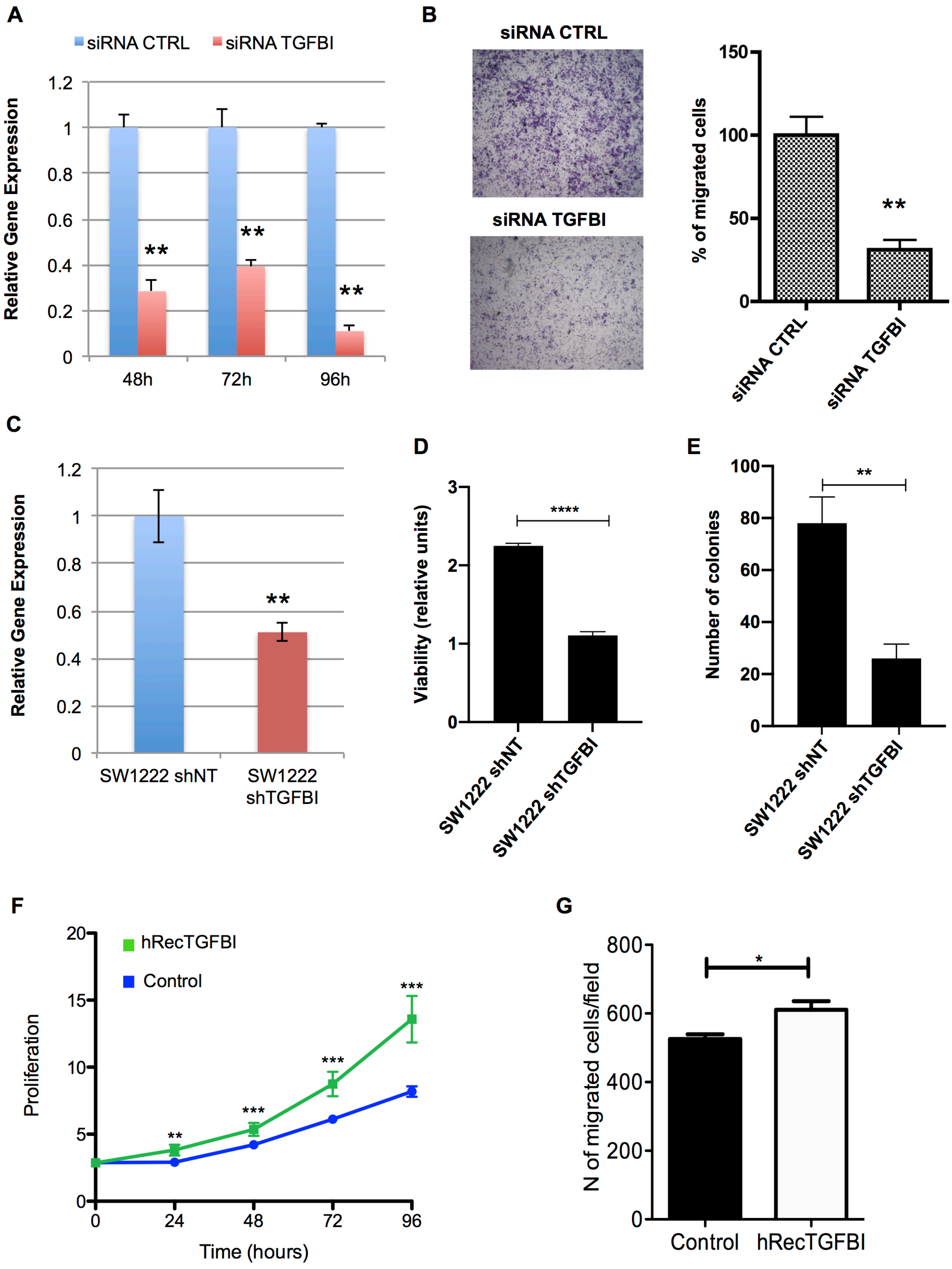

A
siRNA CTRL
siRNA TGFBI
Relative Gene Expression
1.2
1
0.8
0.6
0.4
0.2
0
**
**
**
48h
72h
96h
B
siRNA CTRL
siRNA TGFBI
% of migrated cells
150
100
50
0
**
siRNA CTRL
siRNA TGFBI
C
Relative Gene Expression
1.2
1
0.8
0.6
0.4
0.2
0
**
SW1222 shNT
SW1222 shTGFBI
D
Viability (relative units)
3
2
1
0
****
SW1222 shNT
SW1222 shTGFBI
E
Number of colonies
100
80
60
40
20
0
**
SW1222 shNT
SW1222 shTGFBI
F
hRecTGFBI
Control
Proliferation
20
15
10
5
0
**
***
***
***
0
24
48
72
96
Time (hours)
G
N of migrated cells/field
800
600
400
200
0
*
Control
hRecTGFBI

Figure S4

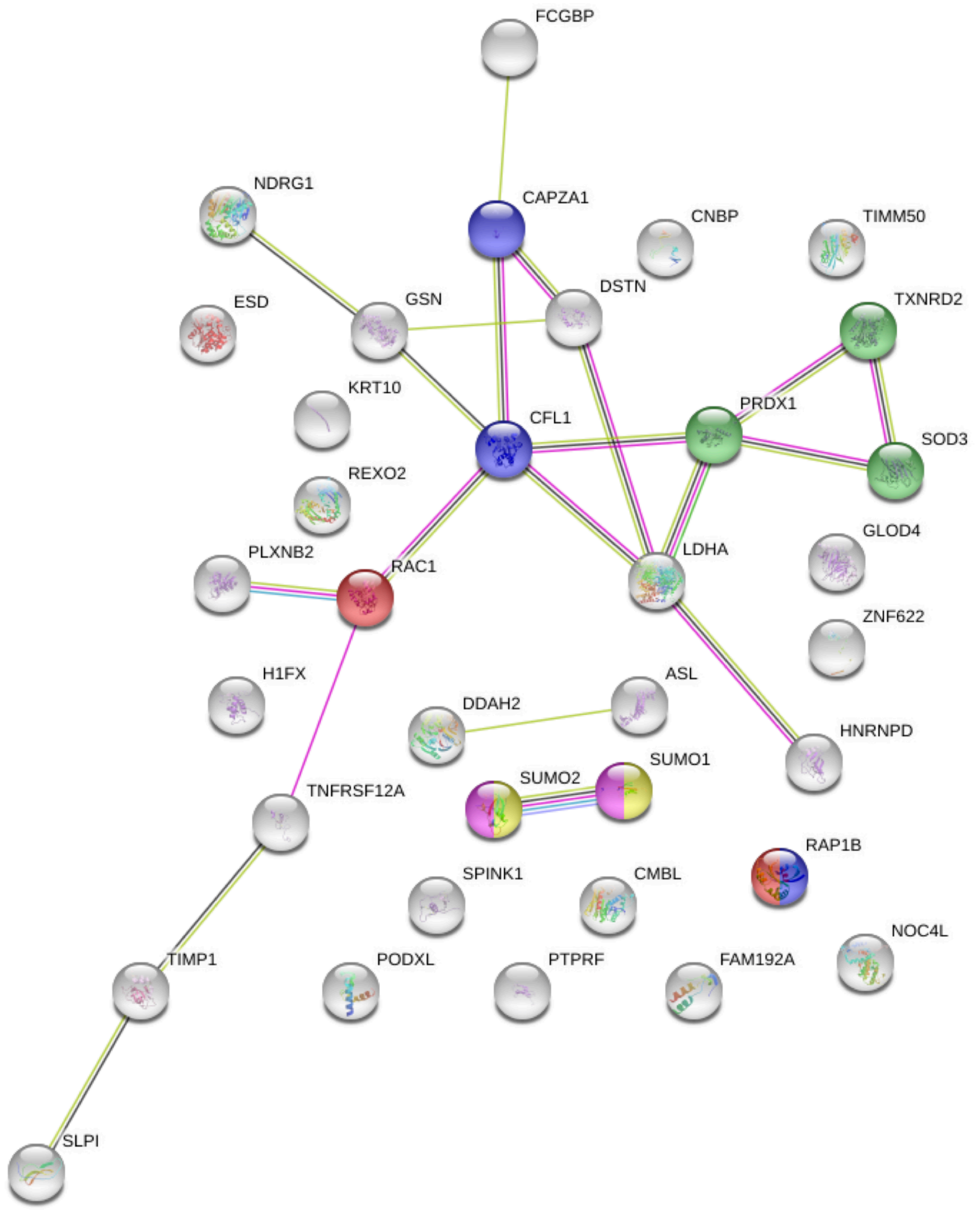

Reactome Pathways

pathway	*description*	*count in gene set*	*false discovery rate*
HSA-8950505	Gene and protein expression by JAK-STAT signaling after In...	3 of 38	0.0093
HSA-8875555	MET activates RAP1 and RAC1	2 of 10	0.0093
HSA-3299685	Detoxification of Reactive Oxygen Species	3 of 35	0.0093
HSA-3065679	SUMO is proteolytically processed	2 of 5	0.0093
HSA-3065678	SUMO is transferred from E1 to E2 (UBE2I, UBC9)	2 of 6	0.0093

Figure S5

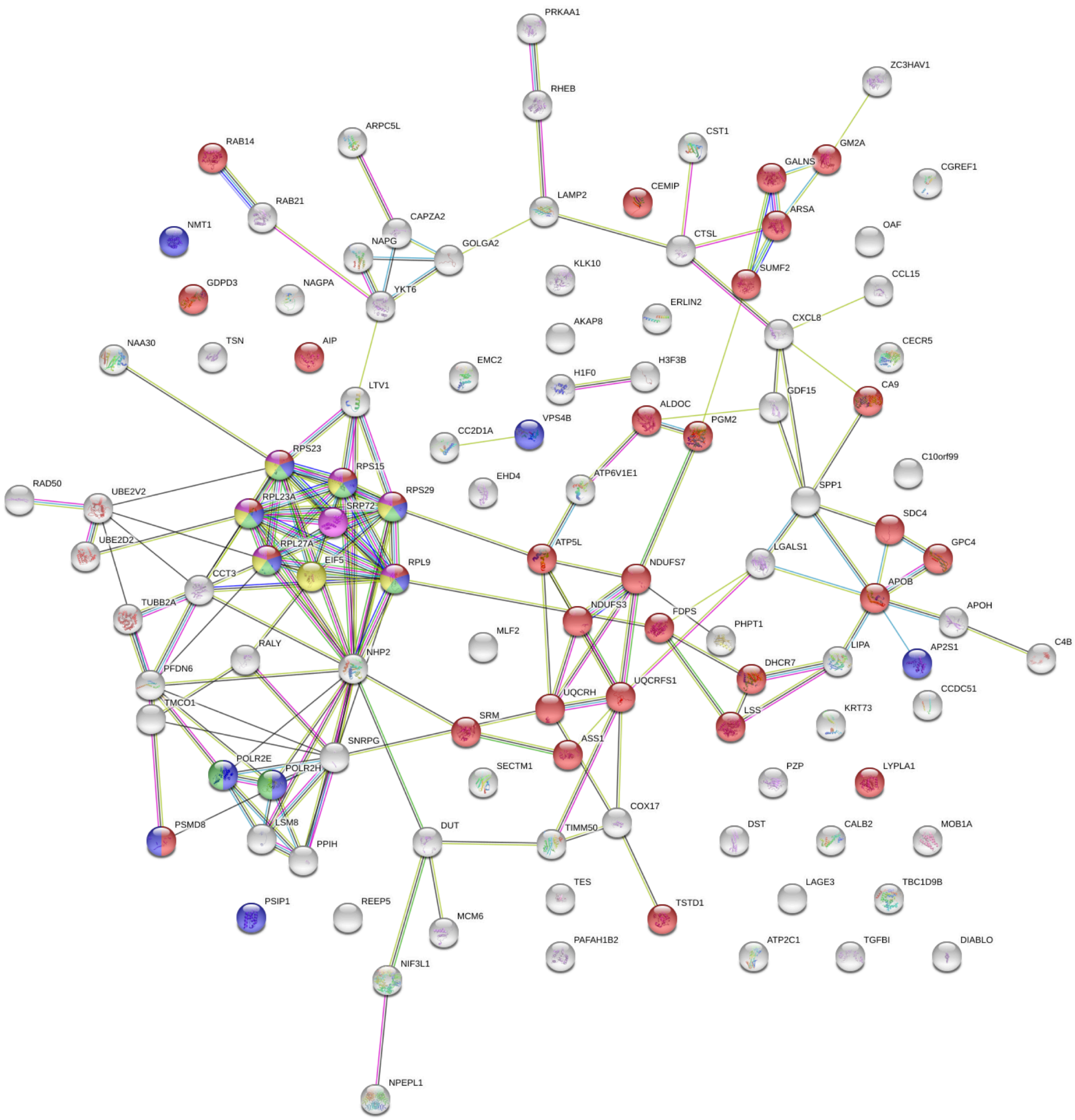

Reactome Pathways

pathway	*description*	*count in gene set*	*false discovery rate*
HSA-1430728	Metabolism	33 of 2032	1.68e-05
HSA-5663205	Infectious disease	13 of 363	7.03e-05
HSA-168273	Influenza Viral RNA Transcription and Replication	8 of 128	0.00022
HSA-72706	GTP hydrolysis and joining of the 60S ribosomal subunit	7 of 108	0.00042
HSA-1799339	SRP-dependent cotranslational protein targeting to membr...	7 of 109	0.00042

Figure S6

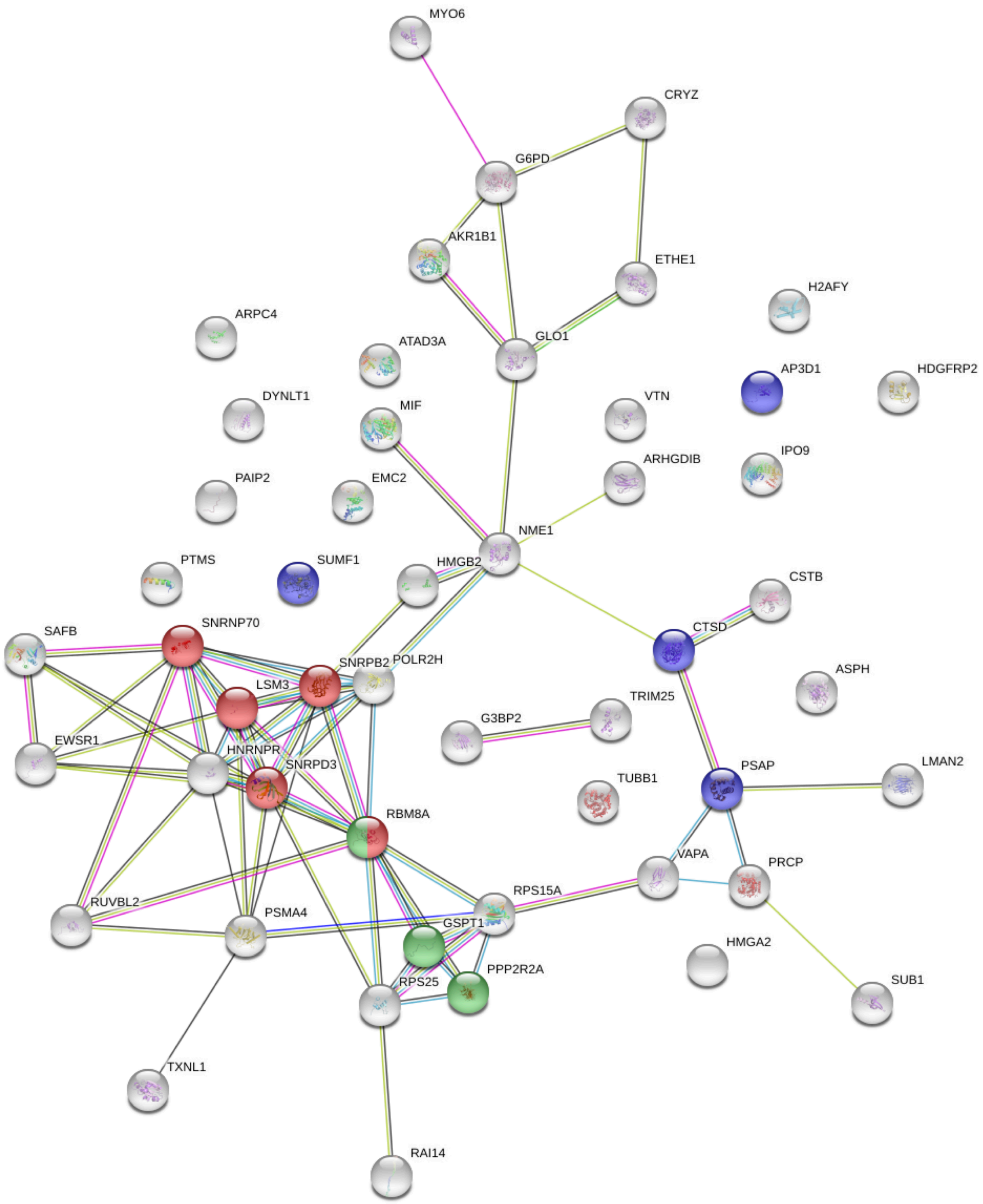

KEGG Pathways

pathway	*description*	*count in gene set*	*false discovery rate*
hsa03040	Spliceosome	5 of 130	0.0018
hsa04142	Lysosome	4 of 123	0.0112
hsa03015	mRNA surveillance pathway	3 of 89	0.0400

Figure S7

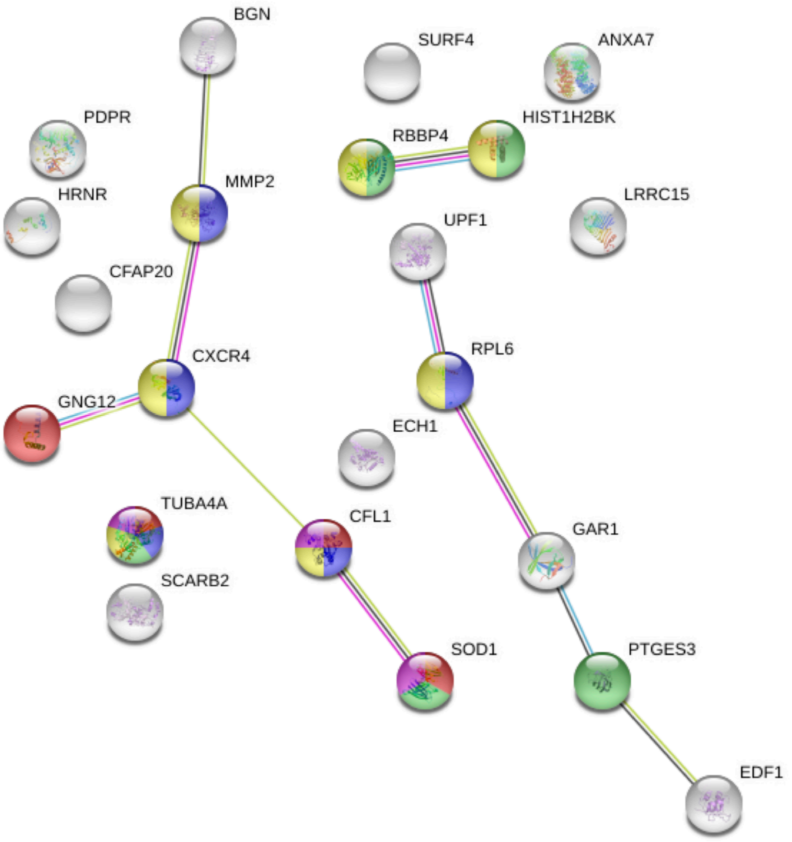

Reactome Pathways			
pathway	*description*	*count in gene set*	*false discovery rate*
HSA-76002	Platelet activation, signaling and aggregation	4 of 256	0.0181
HSA-422475	Axon guidance	5 of 541	0.0181
HSA-2262752	Cellular responses to stress	5 of 384	0.0181
HSA-1266738	Developmental Biology	7 of 1023	0.0181
HSA-114608	Platelet degranulation	3 of 125	0.0192

Figure S8

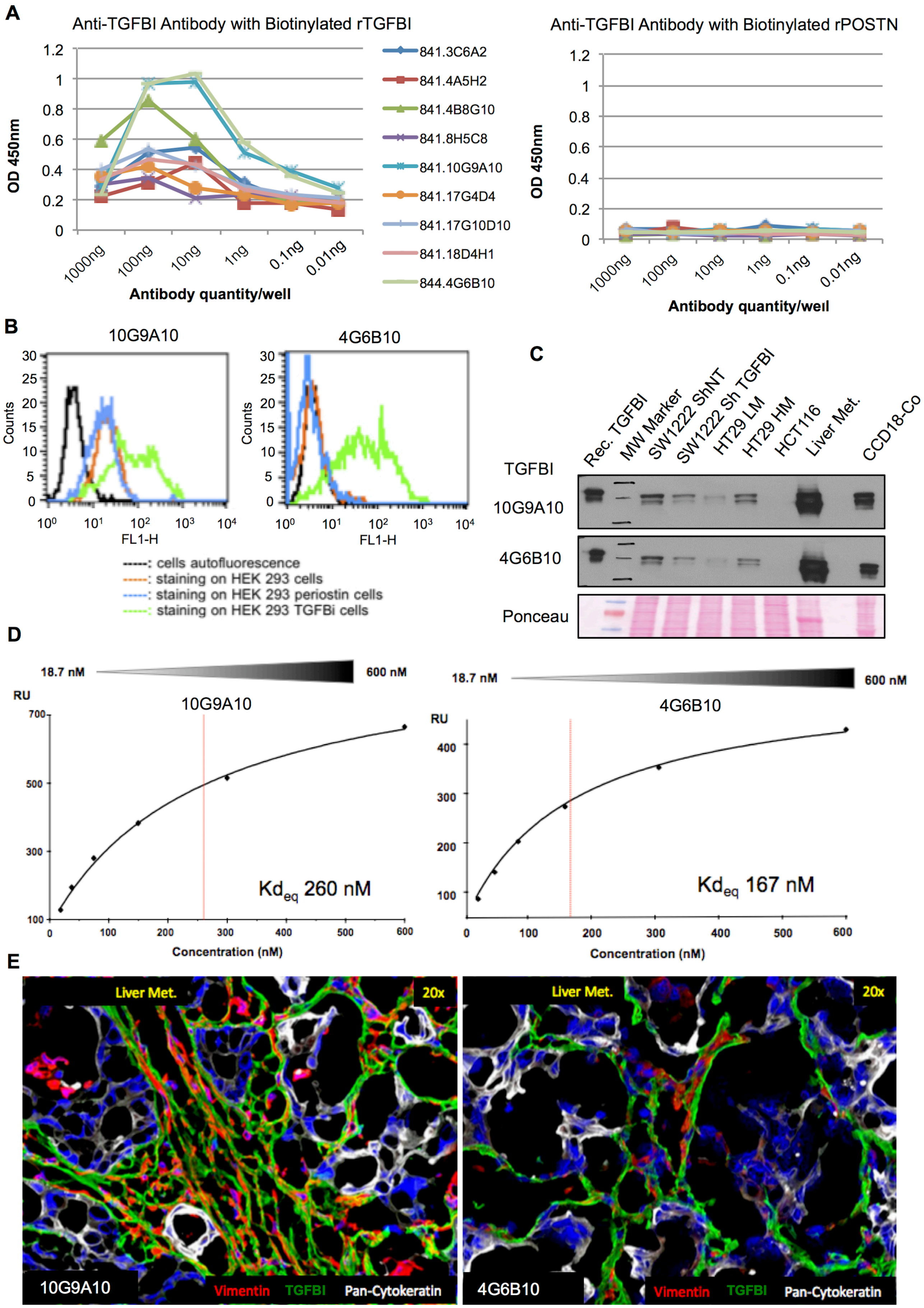
A
Anti-TGFBI Antibody with Biotinylated rTGFBI
OD 450nm
Antibody quantity/well
1000ng
100ng
10ng
1ng
0.1ng
0.01ng
841.3C6A2
841.4A5H2
841.4B8G10
841.8H5C8
841.10G9A10
841.17G4D4
841.17G10D10
841.18D4H1
844.4G6B10
Anti-TGFBI Antibody with Biotinylated rPOSTN
B
10G9A10
4G6B10
Counts
FL1-H
cells autofluorescence
staining on HEK 293 cells
staining on HEK 293 periostin cells
staining on HEK 293 TGFBi cells
C
Rec. TGFBI
MW Marker
SW1222 ShNT
SW1222 Sh TGFBI
HT29 LM
HT29 HM
HCT116
Liver Met.
CCD18-Co
TGFBI
10G9A10
4G6B10
Ponceau
D
18.7 nM
600 nM
RU
10G9A10
Kd$_{eq}$ 260 nM
Concentration (nM)
4G6B10
Kd$_{eq}$ 167 nM
E
Liver Met.
20x
10G9A10
4G6B10
Vimentin
TGFBI
Pan-Cytokeratin
